# Recent Advances in Paper-Based Microfluidic Devices for Heavy Metal Ion Detection: A Review

**DOI:** 10.3390/mi17070780

**Published:** 2026-06-26

**Authors:** Jianqin Xu, Xinyuan Ma, Zhiping Li, Tingting Zhou, Yanshuang Wang, Jianyu Zhu

**Affiliations:** 1 School of Basic Medical Sciences, Beihua University, Jilin City 132013, China; 2 School of Stomatology, Shanxi Medical University, Taiyuan 030001, China; 3 Jilin City People’s Hospital, Jilin City 132011, China; 4 College of Medical Technology, Beihua University, Jilin City 132013, China

**Keywords:** paper-based microfluidics, detection of heavy metal ions, colorimetric detection, fluorescence detection, electrochemical detection, double detection system

## Abstract

Heavy metal ion pollution has emerged as a global issue. These contaminants are not only present in water sources but are also commonly detected in air, soil, food, and consumer products, posing serious risks to ecosystems and human health. Even at very low concentrations, heavy metal ions can exhibit substantial toxicity. Traditional methods for the detection of heavy metal ions typically require complex laboratory equipment and specialized technicians, making them inadequate for rapid on-site monitoring. Microfluidic technology, as an innovative platform capable of precisely controlling and manipulating minute volumes of fluid, has demonstrated enormous potential in analytical chemistry, biomedicine, and environmental monitoring. In the rapidly developing field of microfluidics, paper-based microfluidic platforms have become prominent due to their low cost, straightforward fabrication, and eco-friendly nature, offering powerful tools for the detection of heavy metal ions in diverse samples. This survey consolidates the major advances reported from 2015 to 2025 in utilizing paper-based microfluidic systems for identifying heavy metal ion pollutants in diverse sample types, including air, explosive residues, water sources, herbal supplements, skin-whitening cosmetics, environmental aerosols, urine, soil, gunshot residues, cucumber plants, and food. The review analyzes in detail the principles and applications of detection strategies based on colorimetric methods, fluorescent methods, electrochemical methods, dual-detection systems, and other methods, as well as the role of nanomaterials and selective recognition elements in improving detection sensitivity and specificity. These portable, low-cost, and easy-to-operate detection systems provide viable solutions for environmental and public health monitoring, particularly suitable for resource-limited regions and scenarios requiring rapid detection.

## 1. Introduction

Heavy metal ion contamination has become a major environmental and public health concern worldwide [1,2,3,4,5]. Rapid economic growth and technological development have intensified environmental pollution, making heavy metal contamination one of the most pressing challenges [1,2,3,4,6]. Because heavy metal ions are non-biodegradable, they can accumulate in ecosystems, enter the food chain, and ultimately threaten human health [2,3,4,5,6]. For example, Pb(II) and Hg(II) can damage the nervous system, whereas Cd(II) can accumulate in the kidneys and liver and impair physiological functions [3,5,6]. Therefore, the development of rapid, reliable, and accessible methods for heavy metal ion detection is of great importance for environmental monitoring, food safety, and public health protection [1,6,7].

Conventional methods for heavy metal ion analysis, such as inductively coupled plasma mass spectrometry (ICP-MS), inductively coupled plasma atomic emission spectroscopy (ICP-AES), atomic absorption spectroscopy (AAS), and ultraviolet–visible spectroscopy (UV-Vis), generally provide high sensitivity and accuracy [8,9,10,11]. However, these techniques usually require expensive instrumentation, complex sample preparation, well-controlled laboratory conditions, and trained personnel, which limits their suitability for rapid on-site analysis, especially in resource-limited settings [7,8,9,10,11].

Microfluidic technology has emerged as an attractive alternative for chemical and biological analysis because it enables the manipulation of small volumes of fluids with reduced reagent consumption, rapid response, and high integration [12,13]. Among different microfluidic platforms, paper-based microfluidic analytical devices (µPADs) have attracted considerable attention due to their low cost, portability, ease of fabrication, and ability to operate without external pumps through capillary action [13,14,15,16,17]. In these devices, fluids are guided through hydrophilic channels defined by hydrophobic barriers on porous paper substrates [14,15,16,17]. Sample transport is driven primarily by capillary action, allowing liquid to wick spontaneously through different functional zones without external pumps [15,16,17]. As the sample moves through the device, target analytes can undergo pretreatment, reaction, and signal generation in an integrated and portable format [14,15,16,17]. These features make µPADs particularly suitable for point-of-care testing (POCT) and on-site environmental analysis [13,14,15,16,17].

In recent years, µPADs have been increasingly integrated with colorimetric, fluorescence, electrochemical, and hybrid sensing strategies for heavy metal ion detection [13,18,19,20]. In addition, the use of functional materials, signal amplification strategies, and smartphone-assisted readout has further expanded their analytical capability and practical applicability [19,21,22]. Nevertheless, challenges remain in terms of sensitivity in complex matrices, interference from coexisting substances, reagent stability, and standardization for large-scale application [14,15,16,17,23]. These opportunities and challenges together highlight the need for a comprehensive and updated review of recent progress in this field.

**Figure 1:**
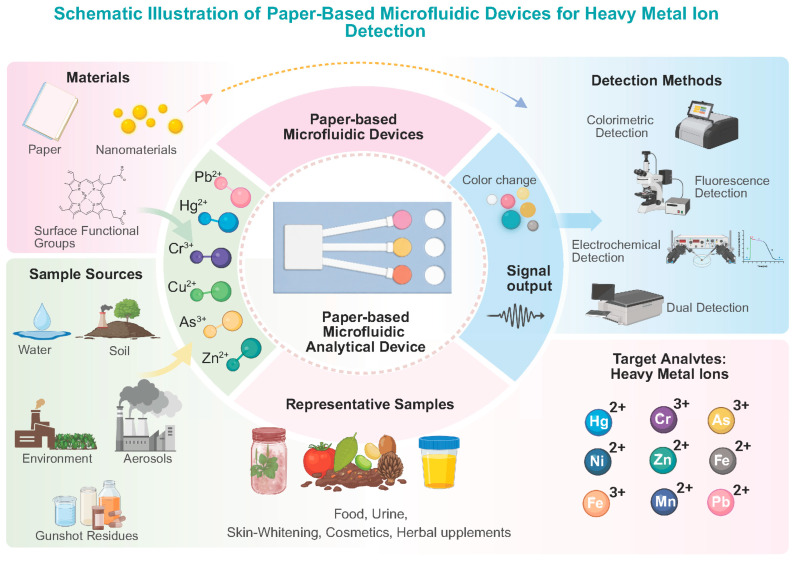
Schematic illustration of the working principle and major detection strategies of paper-based microfluidic devices for heavy metal ion detection. The figure presents an overview of µPAD-based heavy metal ion detection, including functional materials, representative sample matrices, target heavy metal ions, signal generation, and detection strategies. Paper-based microfluidic devices integrate paper substrates, nanomaterials, and surface functional groups with capillary-driven fluid transport to induce signal changes in the sensing region. Representative sample matrices include water, soil, aerosols, gunshot residues, food, urine, cosmetics, and herbal supplements. Representative target ions include Hg(II), Cr(III), As(III), Ni(II), Zn(II), Fe(II)/Fe(III), Mn(II), and Pb(II). The major detection strategies include colorimetric, fluorescence-based, electrochemical, and dual-detection methods. The arrows, colored regions, and dashed circular line indicate the workflow, main functional components, and integrated µPAD platform, respectively. The central capillary-flow-driven paper device schematic was adapted from “Capillary Flow-Driven Microfluidics Combined with a Paper Device for Fast User-Friendly Detection of Heavy Metals in Water” [24], with permission from the copyright holder. Other graphical elements were created in BioRender. JQ, X. (2026) https://BioRender.com/xr6nane.

In this review, we summarize the major advances reported from 2015 to 2025 in µPADs for heavy metal ion detection. We focus on major detection strategies, device design and material-related considerations, representative applications, as well as current challenges and future opportunities in the field. By providing an updated overview of these developments, this review aims to support the further design and practical implementation of µPADs for environmental monitoring and public health protection. The overall working principle and major detection strategies of µPADs for heavy metal ion detection are schematically illustrated in Figure 1.

## 2. Fundamentals of µPADs for Heavy Metal Ion Detection

### 2.1. Device Structure and Working Principles

µPADs have emerged as a promising platform for heavy metal ion detection because of their simple operation, low cost, portability, and suitability for field-based analysis [13,14,15,16,17,23]. In general, µPADs use paper substrates with patterned hydrophilic channels and hydrophobic barriers to guide fluid transport and define reaction zones [14,15,16,17,25,26]. Owing to the intrinsic capillary action of paper, liquid samples can move through the device without the need for external pumps or power sources, making µPADs particularly attractive for POCT and on-site environmental monitoring [15,16,17,23,25,26].

A typical µPAD consists of several functional regions, including a sample introduction zone, a reaction or pretreatment zone, and a detection zone [14,15,16,17,25]. After sample loading, the liquid is distributed through predefined microchannels to different functional areas, where target heavy metal ions interact with chromogenic reagents, fluorescent probes, electrochemical interfaces, or other sensing elements [13,18,19,20,25]. The resulting signal can then be read visually or measured using portable instruments such as smartphones, fluorescence readers, or electrochemical workstations [13,18,19,20,23]. Depending on the analytical design, µPADs can be configured for single-analyte detection, multiplex detection, semi-quantitative screening, or quantitative analysis [14,15,16,17,23].

**Figure 2:**
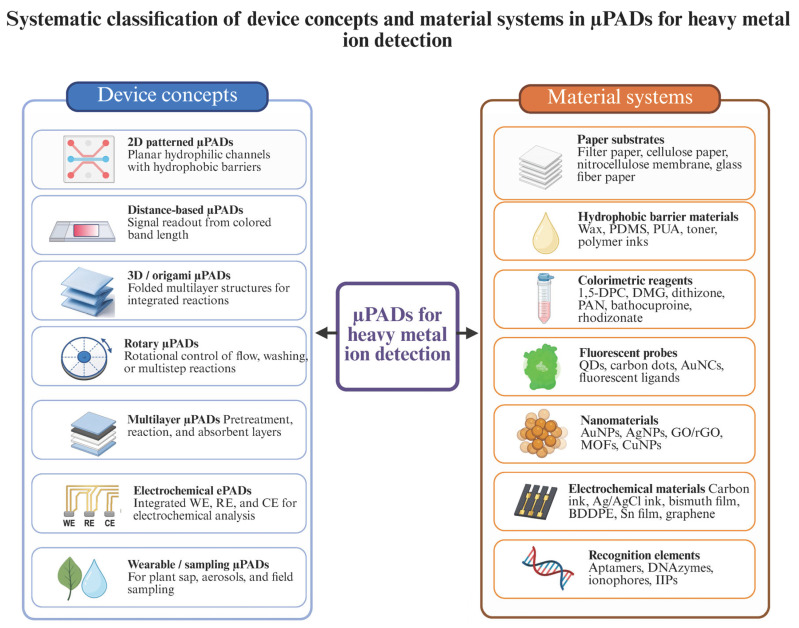
Classification of device concepts and material systems in µPADs for heavy metal ion detection. The device concepts include 2D patterned, distance-based, 3D/origami, rotary, multilayer, electrochemical, and wearable/sampling µPADs, whereas the material systems include paper substrates, hydrophobic barrier materials, colorimetric reagents, fluorescent probes, nanomaterials, electrochemical materials, and recognition elements. Created in BioRender. Xu, J. (2026) https://BioRender.com/047etlb.

With the continuous development of sensing strategies, µPADs have evolved from simple color-change devices into integrated analytical platforms that combine microfluidic manipulation with nanomaterials, biosensing elements, and digital readout systems [14,15,16,17,19,21,22]. These advances have improved analytical performance and broadened the application of µPADs in environmental samples, food products, biological fluids, and consumer products [14,15,16,17,23]. As shown in Figure 2, µPADs for heavy metal ion detection can be broadly summarized from two complementary aspects: device concepts and material systems. Device concepts include two-dimensional (2D) patterned, distance-based, three-dimensional (3D)/origami, rotary, multilayer, electrochemical paper-based analytical devices (ePADs), and wearable/sampling µPADs, whereas material systems include paper substrates, hydrophobic barrier materials, chromogenic reagents, fluorescent probes, nanomaterials, electrochemical materials, and recognition elements.

### 2.2. Substrate Materials and Fabrication Methods

The analytical performance of µPADs is closely related to the properties of the substrate materials and the fabrication methods used to construct the devices [14,15,16,17,23,25,26]. Common substrate materials include filter paper, chromatography paper, cellulose-based paper, and nitrocellulose membranes [14,15,16,17,23]. These materials are widely used because of their low cost, high porosity, favorable capillary flow characteristics, and ease of chemical modification [14,15,16,17,23]. In addition, the introduction of functional groups or nanostructured materials onto paper substrates can further enhance ion capture, selectivity, signal generation, and overall detection sensitivity [22,25].

Various fabrication methods have been developed to create hydrophobic barriers and microfluidic patterns on paper substrates [14,15,16,17,25,26,27,28,29,30,31,32]. Among them, wax printing is one of the most widely used approaches because of its simplicity, low cost, and suitability for rapid prototyping [26]. Other methods, such as inkjet printing [27,28], screen printing [26,29], laser cutting [30], toner laser printing [31], and 3D printing [32], have also been applied to improve patterning precision, structural complexity, and device integration. Different fabrication strategies offer distinct advantages in terms of resolution, scalability, cost, and manufacturing convenience, and the choice of method should therefore depend on the intended application and analytical requirements [14,15,16,17,25,26,27,28,29,30,31,32].

In recent years, the combination of optimized substrate materials and advanced fabrication technologies has greatly promoted the development of µPADs for heavy metal ion detection [14,15,16,17,22,25,26,27,28,29,30,31,32]. These improvements have enabled better fluid control, higher reproducibility, enhanced sensitivity, and more flexible device architectures, thereby supporting the integration of colorimetric, fluorescence, electrochemical, and dual-mode detection strategies on paper-based platforms [18,19,20,21,22]. Nevertheless, further efforts are still needed to achieve standardized, high-resolution, and low-cost manufacturing for large-scale practical applications [14,15,16,17,25,26,27,28,29,30,31,32].

## 3. µPAD-Based Detection Strategies for Heavy Metal Ion Analysis

To date, various detection strategies have been developed for µPADs, including colorimetric, fluorescence, electrochemical, dual-detection, and other emerging methods. Over the past decade, µPADs for heavy metal ion detection have evolved from early proof-of-concept sensors to increasingly integrated analytical platforms. The feasibility of paper-based sensing for heavy metal analysis was first demonstrated by Hossain and Brennan in 2011 [33]. Since then, µPADs have attracted increasing attention because of their low cost, portability, rapid response, and suitability for on-site testing. However, each detection strategy has distinct advantages and limitations in terms of sensitivity, quantitative capability, operational complexity, and instrumentation requirements. Therefore, the following sections compare the major µPAD-based detection methods, focusing on their signal transduction principles, representative technical features, practical applicability, and remaining challenges.

### 3.1. Colorimetric Detection Methods

Colorimetric detection is one of the most widely used strategies for heavy metal ion analysis because of its simplicity, rapid response, low cost, and direct visual readout. In µPAD-based systems, colorimetric signals are generated either through direct color-forming reactions between target metal ions and sensing components or through indirect processes involving conversion, recognition, inhibition, or reagent consumption. Compared with fluorescence-based and electrochemical methods, colorimetric µPADs are generally more suitable for rapid screening and field use, but their quantitative reliability is more susceptible to illumination conditions, reagent distribution, paper background, matrix color, and image acquisition variability.

**Figure 3:**
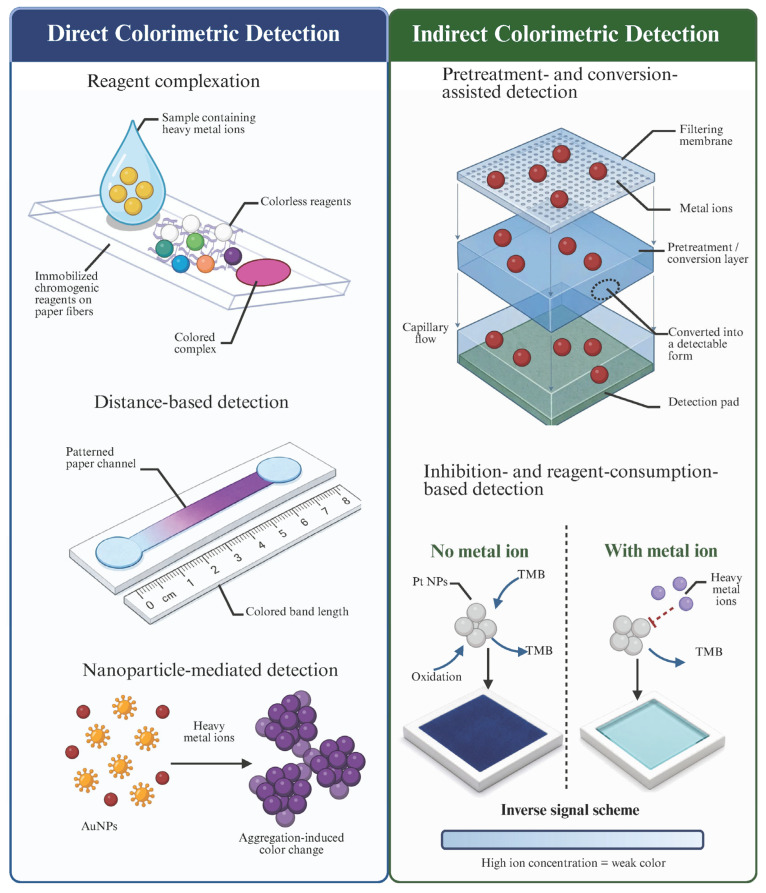
Representative schematic classification of colorimetric µPADs for heavy metal ion detection. Direct colorimetric detection includes chromogenic reagent-based detection, distance-based detection, nanoparticle-mediated detection, multiplex engineered detection, and technology-coupled detection. Indirect colorimetric detection includes conversion-assisted detection, recognition-mediated detection, inhibition-mediated detection, and reagent-consumption and gas-transfer detection. The schematic highlights representative signal-generation mechanisms. Created in BioRender. Xu, J. (2026) https://BioRender.com/qehhp6u.

To avoid simple study-by-study description, this section discusses colorimetric µPADs according to signal-generation mechanisms, device configurations, and readout modes. Direct colorimetric detection mainly includes chromogenic reagent-based detection, distance-based detection, nanoparticle-mediated detection, multiplex engineered detection, and technology-coupled detection. Indirect colorimetric detection includes conversion-assisted detection, recognition-mediated detection, inhibition-mediated detection, and reagent-consumption and gas-transfer detection. The major direct and indirect colorimetric detection strategies used in µPADs for heavy metal ion analysis are schematically summarized in Figure 3.

#### 3.1.1. Direct Colorimetric Detection

Direct colorimetric µPADs generate visible signals through direct interactions between target metal ions and sensing components in the detection region. The resulting color signal can be quantified by visual observation, red–green–blue (RGB) analysis, grayscale extraction, distance measurement, or smartphone-assisted imaging. Based on the signal-generation mechanism and device configuration, direct colorimetric µPADs can be classified into chromogenic reagent-based detection, distance-based detection, nanoparticle-mediated detection, multiplex engineered detection, and technology-coupled detection.

##### Chromogenic Reagent-Based Detection

This category relies on direct coordination or complexation reactions between heavy metal ions and chromogenic reagents, producing visible color changes in the detection zone. The signal is usually quantified by color intensity using scanners, smartphones, or image-processing software. Typical chromogenic systems include Fe(II/III)–phenanthroline, Cr(VI)–1,5-diphenylcarbazide (1,5-DPC), Cu/Zn–1-(2-pyridylazo)-2-naphthol (PAN), Pb/Ba–rhodizonate, and other metal–ligand color-forming reactions.

Early studies mainly demonstrated the feasibility of paper-based colorimetric detection using classical chromogenic reagents. Asano et al. [34] constructed a µPAD for Fe(III) detection in river and tap water samples. The device was fabricated by using a 3D-printed photomask and photolithography to define hydrophilic and hydrophobic regions on paper. In this system, Fe(III) reacted with phenanthroline to form an orange–red complex, and the absorbance at 510 nm was used for quantitative analysis. The same research group [35] further optimized this platform for Cr(VI) detection in water samples. In this assay, 1,5-DPC reacted with Cr(VI) to form a purple complex, and quantitative analysis was performed by measuring RGB color intensity. Ogawa et al. [36] proposed a µPAD for Fe(III) detection in natural spring water. In this system, Fe(III) was first reduced to Fe(II), which then reacted with 1,10-phenanthroline to generate a stable colored complex. Poly(acrylic acid) and acetate buffer were introduced to promote ion-pair formation and improve complex stability. The color intensity was measured using image scanning and ImageJ-based analysis. These representative studies show that classical chromogenic reagent-based µPADs can provide simple and low-cost platforms for Fe and Cr detection in water samples. Their analytical performance mainly depends on the specificity of chromogenic reactions, reagent stability, color uniformity, and reliable optical signal acquisition.

Similar reagent-based systems were later extended to Cu(II), Zn(II), Fe(II), Pb(II), and multi-metal screening in environmental, biological, and forensic samples using PAN, spiroxazine derivatives, sodium rhodizonate, dithizone, aluminon, and other chromogenic reagents. Wu et al. [37] designed a µPAD incorporating a tailored solid-phase extraction (SPE) column for Cu(II) detection in water samples. Sulfonated polystyrene divinylbenzene microspheres were used for sample pretreatment, while PAN served as the chromogenic reagent. The interaction between PAN and Cu(II) caused an orange-to-red color shift. By combining colorimetric analysis with the Euclidean distance method (EDM), Cu(II) concentration was determined according to the degree of color change. Pérez-Rodríguez et al. [38] developed a µPAD for simultaneous monitoring of Cu(II) and Zn(II) in urine samples. In this system, Cu(II) and Zn(II) reacted with PAN under mild conditions to form red and pink chelates, respectively. The color intensity of the metal chelates was quantified by digital image analysis, and a deeper color indicated a higher metal ion concentration. Khunkhong et al. [39] reported a µPAD based on a spiroxazine derivative, 1-benzyl-3,3-dimethylspiro[indoline-2,3′-naphtho [2,1-b][1,4]oxazine] (BSP-SP), for Fe(II) and Pb(II) detection in water samples. Upon ultraviolet (UV) irradiation, BSP-SP underwent structural isomerization to its ring-open form, which acted as a ligand and formed light blue complexes with Fe(II) and Pb(II). The gray intensity of the red channel was measured using smartphone imaging and ImageJ for quantitative analysis. Chabaud et al. [40] developed a µPAD for detecting metal salts in low-explosive residues. Multiple chromogenic reagents were introduced for different metal ions, including sodium rhodizonate for Pb(II) and Ba(II), sodium sulfide for Sb(III), p-aminophenol for Fe(III), ammonium acetate/aluminon for Al(III), dithizone for Zn(II), and xylidyl blue for Mg(II). Metal detection was achieved by observing the corresponding color changes at different concentrations. These studies demonstrate that conventional chromogenic reagent-based µPADs can be extended from single-ion detection to multi-ion screening in water, urine, and forensic samples. The use of different chromogenic reagents enables flexible target selection and simple visual readout, while digital image analysis further supports quantitative assessment. However, reliable application still depends on reagent selectivity, matrix compatibility, color discrimination, and standardized image acquisition.

To improve quantitative performance, recent chromogenic reagent-based µPADs have further incorporated digital imaging and sample pretreatment. Muhammed et al. [41] employed a µPAD for total Cr determination in environmental samples from an industrial zone in Ethiopia. In this system, all Cr species were first oxidized to Cr(VI), followed by reaction with 1,5-DPC to produce 1,5-diphenylcarbazone (DPCO) and Cr(III). Cr(III) further formed a purple complex with DPCO, and the color intensity was recorded using a mobile phone camera and analyzed with ImageJ for quantitative evaluation. Taken together, conventional chromogenic reagent-based µPADs provide a simple and flexible platform for rapid heavy metal screening across water, biological, industrial, and forensic samples. However, their quantitative reliability still depends on reagent stability, pretreatment efficiency, color uniformity, matrix compatibility, and standardized signal acquisition.

##### Distance-Based Detection

Distance-based colorimetric detection converts heavy metal ion concentration into the length of a colored band or precipitate zone along a paper channel. Compared with intensity-based colorimetric detection, this strategy allows direct visual quantification using a printed scale or simple distance measurement, thereby reducing dependence on imaging instruments.

Cai et al. [42] established a distance-based µPAD for Hg(II) detection in whitening cream samples. The device consisted of a circular sample addition zone and a straight reagent-deposition channel defined by wax-printed hydrophobic barriers. Dithizone in alkaline solution reacted with Hg(II) to form a red insoluble complex, which precipitated along the paper channel. The length of the colored precipitate zone was proportional to Hg(II) concentration and was used as the quantitative readout. Buking et al. [43] developed a reaction band-length µPAD for Pb(II) detection in gunshot residues. The device contained a sample storage area, a detection channel, and a waste area, with millimeter scales printed beside the channel. During sample migration, Pb(II) reacted with rhodizonate preloaded in the channel to form a pink insoluble precipitate band. Pb(II) concentration was quantified by directly measuring the length of the colored band. Wisang et al. [44] reported two µPAD designs for Pb(II) detection in wastewater samples using sodium rhodizonate as the chromogenic reagent. Pb(II) reacted with sodium rhodizonate to form a pink Pb–rhodizonate complex. In the first design, Pb(II) concentration was determined by measuring the length of the colored strip in the detection area. In the second design, images of the µPAD were captured with a scanner, and RGB values were analyzed using ImageJ for quantitative assessment.

**Figure 4:**
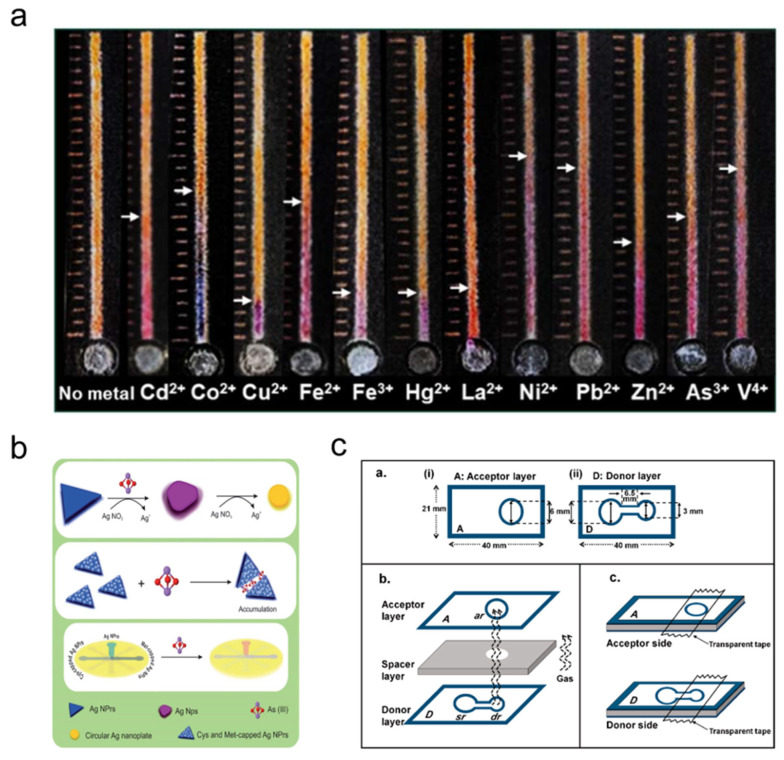
Representative µPADs for the colorimetric detection of heavy metal ions. This figure represents colorimetric detection strategies in µPADs. Panels (**a**,**b**) show direct colorimetric detection methods, including distance-based metallochromic detection and AgNPr-based morphology-mediated colorimetric detection, whereas panel (**c**) shows an indirect gas-separation-assisted colorimetric detection method. The arrows indicate color-development endpoints, morphological transformation, or gas-transfer processes, and the different colors represent visible signal changes generated by heavy metal ion detection. (**a**) Distance-based detection on ion-exchange paper-based microfluidic devices for twelve heavy metal ions using 5-Br-PAPS as the metallochromic reagent. The colored band length is proportional to the analyte concentration. Adapted from Ref. [46], copyright 2024 The Authors, published by the Royal Society of Chemistry under the Creative Commons Attribution-NonCommercial 3.0 Unported Licence. (**b**) Colorimetric detection mechanism for As(III) based on the morphological transformation of silver nanoprisms (AgNPrs). As(III) induces the transformation of triangular AgNPrs into circular nanoplates, resulting in a visible color change from blue to purple. Adapted from Ref. [52], copyright 2022 The Authors, published by the Royal Society of Chemistry under the Creative Commons Attribution-NonCommercial 3.0 Unported Licence. (**c**) Structural design of a three-layer membraneless gas-separation µPAD for equipment-free Hg(II) determination. In panel (**c**), the internal labels a–c are retained from the original figure and indicate different structural/design components of the gas-separation µPAD. Hg(II) is converted into a colorless iodide complex, while residual iodide is oxidized to generate iodine vapor, which diffuses to the acceptor layer and forms a purple triiodide–starch complex for image-based quantification. Adapted from Ref. [72], copyright 2021 The Authors, distributed under the terms of the Creative Commons Attribution 4.0 International License.

Distance-based strategies have also been extended to soil and multi-metal screening. Guan et al. [45] designed an atomic stamp printing-fabricated µPAD for Cu(II) detection in soil samples. Hydrophobic patterns were produced by stamping polydimethylsiloxane (PDMS) onto paper. Cu(II) reacted with sodium diethyldithiocarbamate (DDTC) to form a yellow–brown complex. The device contained an S-shaped detection channel, and Cu(II) concentration was evaluated by measuring the length of the colored band along the channel. ImageJ-based gray value analysis was also used for signal quantification. Manmana et al. [46] developed a distance-based µPAD for rapid visual quantification of heavy metals in herbal supplements and cosmetics (Figure 4a). In this system, 2-(5-Bromo-2-pyridylazo)-5-[N-propyl-N-(3-sulfopropyl)amino]phenol (5-Br-PAPS) was adsorbed onto ion-exchange filter paper and reacted with different metal ions to generate visible color changes. The length of color development along the detection channel corresponded to metal ion concentration and could be read directly using a printed scale beside the channel.

Distance-based colorimetric µPADs provide a simple and instrument-free readout format by converting color development or precipitate formation into a measurable distance. This strategy is useful for field analysis because the signal can be interpreted visually without complex instruments. However, reliable quantification still depends on uniform capillary flow, stable reagent distribution, clear color-boundary formation, and appropriate control of sample extraction and matrix effects.

##### Nanoparticle-Mediated Detection

Nanoparticle-mediated colorimetric detection relies on optical changes in plasmonic nanoparticles, mainly gold nanoparticles (AuNPs) and silver nanoparticles (AgNPs). Target heavy metal ions can induce nanoparticle aggregation, disintegration, etching, or morphological transformation, leading to changes in localized surface plasmon resonance (LSPR) and visible color variation. According to the nanomaterial used, nanoparticle-mediated colorimetric µPADs can be mainly classified into AuNP-based and AgNP-based systems.

AuNP-based µPADs commonly rely on aggregation- or dispersion-induced color changes. Chowdury et al. [47] developed a µPAD incorporating AuNPs functionalized with α-lipoic acid and thioguanine for As(III) detection in groundwater. In the absence of As(III), the nanosensors showed a red color, whereas interaction with As(III) induced nanoparticle aggregation and changed the color to black. The red intensity ratio of the detection area was analyzed using ImageJ for quantitative assessment. Abdollahiyan et al. [48] reported a µPAD based on cysteine-modified AuNPs (CysA@AuNPs) for Hg(II) and Cu(II) detection. Hg(II) induced partial aggregation of CysA@AuNPs through its strong affinity for cysteine, resulting in a color shift from burgundy to pink. In contrast, Cu(II) caused depolymerization of CysA@AuNPs through chelation with cysteine and methionine, leading to a color change from pink to ruby red. The concentrations of Hg(II) and Cu(II) were quantified by monitoring changes in UV-Vis absorption spectra.

AgNP-based µPADs generally produce colorimetric signals through aggregation, disintegration, etching, or morphological transformation. Rasheed et al. [49] designed a sensor using metronidazole-functionalized AgNPs (MTZ-AgNPs) for Pb(II) detection in environmental samples. Pb(II) induced MTZ-AgNP aggregation, causing a visible color transition from yellow to red and a red shift in the LSPR absorption peak. After integration into a µPAD, Pb(II) was quantified by measuring both the color change and the distance or height change in the reaction area. Meelapsom et al. [50] developed an inkjet-printed µPAD using unmodified AgNPs for Hg(II) detection in water samples. Hg(II) induced AgNP disintegration, resulting in a color change from dark to bright yellow. The RGB signal was converted into digital values and analyzed using image-processing software for quantitative detection. Fu et al. [51] reported a µPAD-based AgNP system for Hg(II) detection in food samples, in which AgNPs changed from yellow to colorless after interaction with Hg(II). Hg(II) concentration was determined by converting the color change into RGB-based digital signals. Saadati et al. [52] developed a µPAD based on AgNP morphological transformation for As(III) detection in urine samples (Figure 4b). As(III) promoted silver atom deposition on AgNP surfaces, changing their morphology from triangular to round and producing a color change from blue to purple. The signal was determined visually or by image analysis software.

Nanoparticle-mediated colorimetric µPADs improve visual sensitivity by converting heavy metal–nanoparticle interactions into distinct color changes. AuNP-based systems mainly rely on aggregation, dispersion, or surface-functionalized recognition, whereas AgNP-based systems often involve aggregation, disintegration, etching, or morphological transformation. These strategies provide strong visual signals and are suitable for portable detection, but reliable application still depends on nanoparticle stability, surface modification reproducibility, ionic strength, pH, matrix effects, and standardized optical signal acquisition.

##### Multiplex Engineered Detection

Multiplex engineered detection improves analytical throughput and fluid control by integrating multiple detection zones, pretreatment regions, multilayer structures, rotary valves, or capillary-driven flow designs into a single paper-based platform. Compared with simple single-zone devices, these systems are particularly useful for simultaneous multi-ion detection, metal speciation, reagent confinement, and controlled sample transport.

Sharifi et al. [53] designed a 3D origami µPAD incorporating a polyvinyl chloride (PVC) film for Cu(II) detection in water samples. The device consisted of a sample area, multiple preprocessing areas, and a detection area. Cu(II) formed a blue complex with chrome azurol S or catechol violet, and the color intensity was quantified by scanning the device and analyzing RGB values using ImageJ. Muhammed et al. [54] developed a two-channel µPAD for chromium speciation in water samples. The left channel was used for direct Cr(VI) determination based on the colorimetric reaction between Cr(VI) and 1,5-DPC. The right channel was used for total Cr determination, in which Cr(III) was first oxidized online to Cr(VI) in a Ce(IV)-containing pretreatment zone and then detected using the same 1,5-DPC colorimetric reaction. The Cr(III) concentration was calculated by subtracting the Cr(VI) signal from the total Cr signal.

Multi-zone and chemically functionalized µPADs have also been used for parallel detection of different metal ions. Xiong et al. [55] developed a µPAD with one central region, ten reaction regions, and ten detection regions for Fe(III) and Ni(II) detection. Fe(III) reacted with 1,10-phenanthroline to produce an orange complex, whereas Ni(II) reacted with dimethylglyoxime (DMG) to form a pink complex. The color intensity was quantified by converting device images into grayscale values using ImageJ. Li et al. [56] designed a periodic-table-style µPAD for simultaneous Cu(II), Cr(VI), and Ni(II) detection. Cu(II), Cr(VI), and Ni(II) reacted with bathocuproine, 1,5-DPC, and DMG, respectively, producing visible color changes that revealed the corresponding element symbols on the device. Devadhasan et al. [57] reported a chemically functionalized µPAD in which amine, carboxyl, and sulfhydryl groups were introduced onto chromatographic paper to immobilize different chromogenic reagents. Ni(II), Cr(VI), and Hg(II) were detected using DMG, 1,5-DPC, and millethione, respectively, and RGB intensities were analyzed using ImageJ for quantitative assessment. Kamnoet et al. [58] developed a wax-printed µPAD containing multiple pretreatment and detection areas for simultaneous detection of Cu(II), Co(II), Ni(II), Hg(II), and Mn(II). The corresponding chromogenic reagents included bathocuproine, 4-(2-pyridinazo)resorcinol (PAR), DMG, and dithizone, and the color intensities were obtained from scanned images using ImageJ.

Flow-control structures, such as rotary and capillary-driven devices, have further improved the operation of multiplex colorimetric µPADs. Sun et al. [59] designed a rotary µPAD for simultaneous detection of Ni(II), Cu(II), and Cr(VI) in environmental water. The rotary valve controlled the connection between the detection zone and the fluid channel, while Ni(II), Cu(II), and Cr(VI) were detected using DMG, bathocuproine, and 1,5-DPC, respectively. Punnoy et al. [60] developed a capillary-flow-driven, single-dip dual-sided µPAD for Ni(II), Fe(III), and Cu(II) detection in drinking water. Ni(II), Fe(III), and Cu(II) reacted with DMG, bathophenanthroline, and bathocuproine, respectively, and the signal was quantified using smartphone-based digital image colorimetry through the green channel, with a control zone used to normalize lighting variations. Aryal et al. [24] reported a capillary-driven µPAD containing pretreatment, detection, and waste zones for Ni(II), Cu(II), and Fe(III) analysis. The color changes produced by DMG, bathocuproine, and bathophenanthroline were captured using a smartphone camera, and grayscale values were analyzed with ImageJ.

Collectively, these studies show that multiplex and structurally engineered colorimetric µPADs can expand direct colorimetric detection from single-analyte assays to multi-ion and speciation analysis. Multilayer, rotary, capillary-driven, and multi-zone structures improve reagent localization, sample distribution, flow control, and parallel detection capability. However, their reliable application still depends on reagent compatibility, channel-to-channel reproducibility, accurate layer alignment, uniform capillary flow, and effective control of cross-interference among different detection zones.

##### Technology-Coupled Detection

Technology-coupled colorimetric platforms extend the capability of conventional colorimetric µPADs by integrating auxiliary imaging devices, molecular recognition systems, automated fabrication tools, field-sampling platforms, or instrumental confirmation methods. These systems retain the visual readability of colorimetric detection while improving image acquisition, target recognition, sample collection, or quantitative analysis.

Imaging-assisted platforms have been developed to improve colorimetric signal acquisition and visual interpretation. Vaishampayan et al. [61] designed a µPAD integrated with a 3D-printed imaging box for Cr(VI) detection in water samples. Cr(VI) reacted with 1,5-DPC under acidic conditions to form a purplish-red complex, and the color intensity was analyzed using ImageJ under uniform light-emitting diode (LED) illumination provided by the imaging box. Gabhane et al. [62] developed a Foldscope-integrated µPAD for As(III) detection in water. In this system, As(III) induced aggregation of functionalized AuNPs, causing an LSPR shift and a visible color transition from red to blue. The color change was recorded using a smartphone camera, while the Foldscope was used to magnify the detection disc and enhance visual observation. Yao et al. [63] developed a µPAD integrated with an automated dispensing machine for large-scale Cr(VI) detection in industrial wastewater. Cr(VI) reacted with 1,5-DPC to form a purple–red complex, and color intensity was quantified using ImageJ under LED illumination within an enclosed dark chamber.

Molecular-recognition- and app-assisted platforms provide another route for improving specificity and digital quantification. Zhang et al. [64] developed a CRISPR-Cas12a-based µPAD for Pb(II) detection in water samples. In the absence of Pb(II), the CRISPR-Cas12a system cleaved single-stranded DNA (ssDNA) surrounding AuNPs, preventing color development. In the presence of Pb(II), G-quadruplex formation inhibited CRISPR-Cas12a activation, allowing uncleaved ssDNA-AuNPs to be captured by DNA nanoflowers and turn the detection region red. The red/green (R/G) ratio of the detection area was analyzed using the SmartIons app for quantitative assessment.

Sampling- and instrument-coupled platforms have expanded colorimetric µPADs beyond conventional water analysis. Sun et al. [65] developed a µPAD using graphene oxide nanosheets to collect airborne particulate matter samples via a drone-carried sampler. Fe(II), Cu(II), and Ni(II) reacted with 1,10-phenanthroline, dithiourea, and DMG, respectively, to form colored complexes, and smartphone-captured images were analyzed using a precalibrated standard curve. Sun et al. [66] further introduced a portable µPAD method combined with unmanned aerial vehicle (UAV)-based sampling and smartphone imaging for Co(II), Cu(II), Fe(III), Mn(II), Cr(VI), and Ni(II) detection in airborne particulate matter. Metal concentrations were quantified by analyzing grayscale changes in smartphone images. Ma et al. [67] developed a foldable laser-induced breakdown spectroscopy (LIBS)-assisted paper-based microfluidic analytical device (LaPAD) for Cu(II) and Mn(II) detection in water samples. The colorimetric module provided rapid semi-quantitative screening using commercial test strips, while the concentration-gradient module generated standard solutions for LIBS-based quantitative analysis.

Technology-coupled colorimetric platforms improve the practical utility of µPADs by enhancing imaging standardization, molecular recognition, sample collection, automated fabrication, or quantitative confirmation. However, these systems often require additional accessories or instruments, which may increase device complexity and reduce the simplicity of fully paper-based assays. Future development should therefore focus on balancing analytical performance with portability, operational simplicity, and field robustness.

#### 3.1.2. Indirect Colorimetric Detection

Indirect colorimetric detection is used when heavy metal ions do not directly produce a strong visible signal with chromogenic reagents or when additional conversion, recognition, inhibition, or reagent-consumption steps are required to generate a measurable color response. Compared with direct colorimetry, indirect colorimetric µPADs offer greater flexibility for metal speciation, signal regulation, and complex sample analysis, but they usually involve more reaction steps and require stricter control of reaction conditions. Based on the way in which the target ion indirectly modulates the color-forming process, indirect colorimetric µPADs can be classified into conversion-assisted detection, recognition-mediated detection, inhibition-mediated detection, and reagent-consumption and gas-transfer detection.

##### Conversion-Assisted Detection

Conversion-assisted chromogenic detection relies on pretreatment steps such as oxidation, reduction, extraction, or separation to convert target metal ions into detectable forms before color development. This strategy is particularly useful for metal speciation and multiplex analysis, where different oxidation states or coexisting ions need to be distinguished before chromogenic reactions.

Li et al. [68] designed a dual-layer 3D µPAD for the simultaneous determination of Fe(III), Ni(II), Cr(VI), Cu(II), Al(III), and Zn(II) in environmental samples. The upper layer served as the pretreatment layer, and the lower layer functioned as the colorimetric detection layer. After sample introduction, the solution was distributed into multiple channels for pretreatment and color development. Fe(III) was reduced to Fe(II), which reacted with 1,10-phenanthroline to form an orange–red complex. Cu(II) was reduced to Cu(I), which reacted with bathocuproine to form a yellow complex. Ni(II), Cr(VI), Al(III), and Zn(II) were detected through their reactions with DMG, 1,5-DPC, chrome azurol S, and dithizone, respectively. Color images of the detection layer were captured using a smartphone, and ImageJ was used to analyze ΔGray and ΔE values for quantitative assessment. Tabani et al. [69] integrated double-gel electromembrane extraction with a µPAD for simultaneous Cr(III) and Cr(VI) determination in water samples. In this system, Cr(III) migrated to the cathodic receptive phase, whereas Cr(VI) migrated to the anodic receptive phase. Cr(VI) reacted with diphenylcarbazide to form a purple complex, while Cr(III) was first oxidized to Cr(VI) and then detected using the same chromogenic reaction. The color intensity on the µPAD was analyzed using ImageJ by measuring the green-channel intensity for quantitative determination of the two chromium species.

Conversion-assisted detection extends colorimetric µPADs from simple total-ion analysis to metal speciation and multiplex detection. By integrating reduction, oxidation, extraction, or separation steps before color development, this strategy enables target discrimination in complex samples. However, reliable performance still depends on pretreatment efficiency, pH control, reagent compatibility, fluid distribution, and accurate colorimetric signal acquisition.

##### Recognition-Mediated Detection

Recognition-mediated nanoparticle colorimetry combines specific recognition elements with the optical response of plasmonic nanoparticles. In these systems, target metal ions regulate nanoparticle aggregation or dispersion through aptamer binding or surface interactions, resulting in visible color changes and LSPR shifts.

Fakhri et al. [70] developed an aptamer-based µPAD for Pb(II) detection in water samples. In this platform, Pb(II) recognition by the aptamer regulated the salt-induced aggregation of AuNPs. In the presence of Pb(II), AuNPs aggregated after interaction with NaCl, leading to an LSPR response and a visible color shift from red to purple. The color change intensity was quantified using UV-Vis and ImageJ-based image analysis.

This strategy introduces molecular recognition into nanoparticle-mediated colorimetric detection and enables target-regulated aggregation-based signal generation. However, reliable application still depends on aptamer stability, salt-induced aggregation control, nanoparticle dispersion, and substrate-dependent signal variation.

##### Inhibition-Mediated Detection

Inhibition-mediated colorimetry uses the inhibitory effect of heavy metal ions on specific color-forming reactions to generate a measurable signal. In this strategy, the target ion does not directly produce a colored product but suppresses a catalytic or chromogenic reaction, resulting in a decrease in color intensity.

Chen et al. [71] developed a µPAD for Hg(II) detection in water samples. In this system, platinum nanoparticles catalyzed the oxidation of 3,3′,5,5′-tetramethylbenzidine (TMB), producing a blue color in the detection zone. The presence of Hg(II) inhibited this oxidation process, leading to a decrease in blue intensity. The Hg(II) concentration was therefore determined based on the inverse relationship between Hg(II) level and color intensity, which could be visually observed or quantified from the detection area.

This inhibition-based strategy expands indirect colorimetric detection by converting metal-induced reaction suppression into a measurable signal. However, because the signal decreases with increasing analyte concentration, reliable quantification requires careful control of reaction time, catalytic activity, reagent stability, and visual or image-based signal interpretation.

##### Reagent-Consumption and Gas-Transfer Detection

Reagent-consumption and gas-transfer colorimetry is based on the consumption of a key reagent by the target metal ion, followed by secondary color development through vapor generation, migration, and reaction in a separate detection zone. This strategy converts metal-ion-induced reagent consumption into an inverse colorimetric signal.

Nashukha et al. [72] developed a µPAD for Hg(II) detection in contaminated soil. In this system, Hg(II) reacted with iodide ions to form tetraiodomercurate complexes (Figure 4c). The remaining iodide ions were then oxidized under acidic conditions to generate iodine vapor, which migrated to the detection zone and reacted with starch to form a purple triiodide–starch complex. Because Hg(II) consumed iodide ions, the Hg(II) concentration was inversely correlated with the color intensity of the triiodide–starch complex. The final color signal was quantified using image analysis software.

This reagent-consumption and gas-transfer strategy provides an indirect route for Hg(II) detection by linking iodide consumption, iodine vapor transfer, and starch-based color development. Because the signal decreases with increasing Hg(II) concentration, reliable quantification requires careful control of iodide concentration, oxidation conditions, vapor transport, reaction time, and matrix effects.

In summary, colorimetric µPADs provide the simplest and most visually accessible strategy for heavy metal ion detection, but their performance varies substantially among different signal-generation mechanisms. Chromogenic reagent-based systems are easy to implement and suitable for common metal ions, but they are limited by reagent selectivity, matrix interference, and color discrimination. Distance-based devices reduce the dependence on optical instruments by converting concentration into band length, whereas their accuracy depends strongly on uniform capillary flow and clear color-boundary formation. Nanoparticle-mediated systems improve visual sensitivity through LSPR-related color changes, but nanoparticle stability, aggregation control, ionic strength, and pH remain important limitations. Multiplex engineered and technology-coupled platforms further improve throughput, flow control, imaging standardization, and field applicability, but they may increase device complexity and reduce the simplicity of conventional µPADs. Therefore, future colorimetric µPADs should focus on improving reagent immobilization, color uniformity, anti-interference capability, internal calibration, and standardized image analysis. Representative direct and indirect colorimetric µPADs for heavy metal ion detection are summarized in Table 1 and Table 2, respectively.

### 3.2. Fluorescence Detection Methods

Fluorescence detection is another commonly used strategy for heavy metal ion analysis and generally provides higher sensitivity and better signal contrast than simple colorimetric detection. In µPAD-based systems, heavy metal ions can regulate fluorescence signals by quenching, enhancing, recovering, or shifting the emission of fluorescent probes. Therefore, fluorescence-based µPADs are discussed here according to their fluorescence response patterns and signal-generation mechanisms rather than as a simple sequence of individual studies.

**Figure 5:**
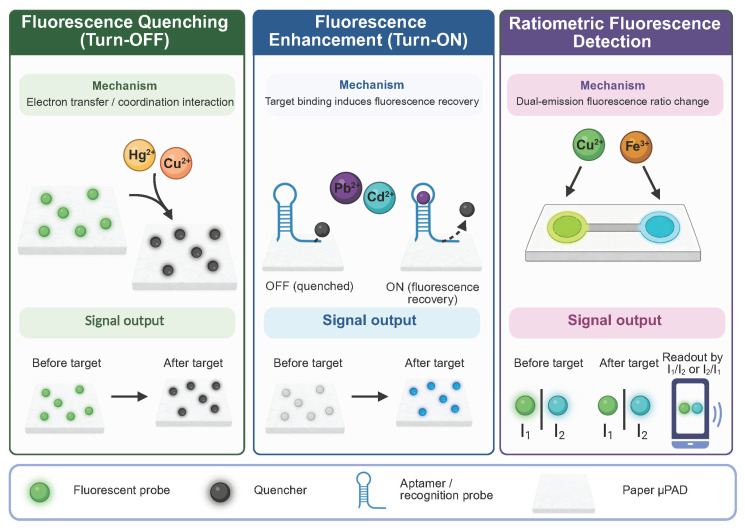
Schematic classification of fluorescence-based µPADs for heavy metal ion detection. Fluorescence-based µPADs can be classified into fluorescence quenching, fluorescence enhancement, and Ratiometric fluorescence detection according to their signal response modes. Fluorescence quenching is based on the decrease or disappearance of fluorescence after target metal ions interact with fluorescent probes through electron transfer, coordination interaction, or surface-related effects. Fluorescence enhancement relies on fluorescence recovery or signal amplification after target binding, quencher displacement, ligand complexation, or recognition-mediated structural changes. Ratiometric fluorescence detection uses dual-emission signals, in which the intensity ratio between two fluorescence channels is used for quantitative analysis and internal signal correction. The arrows indicate target-induced signal changes, including fluorescence quenching, fluorescence recovery, or ratiometric signal response. The colored dots represent fluorescent probes, quenchers, target ions, or fluorescence signal outputs as indicated in the legend. Created in BioRender. JQ, X. (2026) https://BioRender.com/90o69ue.

According to the fluorescence response mode, fluorescence-based µPADs can be broadly classified into fluorescence quenching, fluorescence enhancement, and ratiometric fluorescence detection. Fluorescence quenching provides simple turn-off responses but is susceptible to nonspecific quenching and background interference. Fluorescence enhancement produces turn-on signals through metal–ligand complexation, nucleic acid structure regulation, or strand displacement, but it requires stable probes and controlled reaction conditions. Ratiometric fluorescence improves quantitative reliability by using internal signal correction, although it usually involves more complex probe design and ratio-based image analysis. The main fluorescence response modes and their signal changes are schematically summarized in Figure 5.

#### 3.2.1. Fluorescence Quenching

Fluorescence quenching is based on the reduction in or disappearance of fluorescence intensity after fluorescent probes interact with target metal ions or quencher molecules. According to the quenching mechanism and recognition strategy, fluorescence-quenching µPADs can be classified into direct metal–fluorophore quenching, recognition-regulated switching, and ion-imprinted QD quenching.

##### Direct Metal–Fluorophore Quenching

Direct metal–fluorophore interaction-induced quenching relies on the direct interaction between target metal ions and fluorescent probes, such as gold nanoclusters (AuNCs) or quantum dots (QDs). This interaction can suppress fluorescence through electron transfer, surface-state changes, or coordination interactions, resulting in a turn-off fluorescence response.

**Figure 6:**
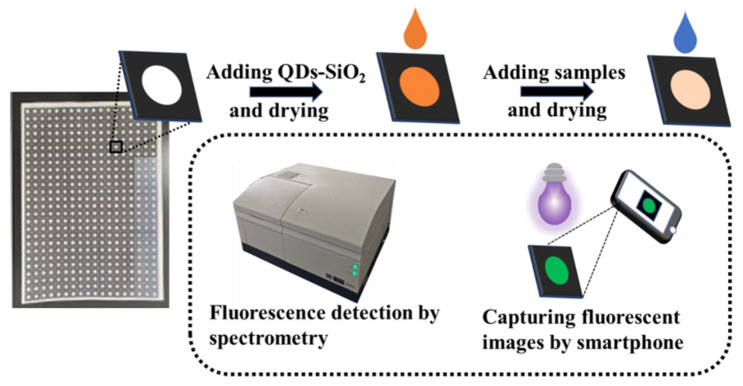
Representative µPAD for fluorescence quenching detection of Hg(II). This figure represents a QD-based fluorescence quenching method using CdTe QDs–SiO_2_-modified paper chips. The QDs–SiO_2_ solution is added to the detection zone and dried, followed by sample addition and fluorescence signal acquisition. The fluorescence signal can be measured by spectrometry or captured by smartphone imaging for quantitative analysis. Adapted from Ref. [74], copyright 2023 The Authors, distributed under the terms of the Creative Commons Attribution 4.0 International License.

Fang et al. [73] developed a µPAD using bovine serum albumin-stabilized gold nanoclusters (Au-BSA NCs) for Cu(II) detection in environmental water samples. The device consisted of a sample injection area, a detection zone, and an absorbent pad. Au-BSA NCs emitted red fluorescence under UV light. In the presence of Cu(II), the fluorescence of Au-BSA NCs was quenched, and the quenching distance along the strip was proportional to Cu(II) concentration. The fluorescence signal was visually observed under UV light, and the distance-based fluorescence response was used for quantitative analysis. Han et al. [74] developed a µPAD using CdTe QD-modified silica nanoparticles (QDs–SiO_2_) for Hg(II) detection in environmental water samples (Figure 6). The QDs–SiO_2_ complex was immobilized in the detection area of the paper device. CdTe QDs emitted fluorescence under UV excitation, and Hg(II) selectively quenched the fluorescence through electron transfer. The fluorescence intensity decreased with increasing Hg(II) concentration. During detection, the fluorescence signal was captured using a smartphone camera, and the gray intensity was analyzed using ImageJ for quantitative determination of Hg(II).

Direct metal–fluorophore interaction-induced quenching provides a straightforward fluorescence turn-off strategy for heavy metal ion detection in µPADs. AuNC- and QD-based probes convert metal–probe interactions into measurable decreases in fluorescence intensity or quenching distance. However, reliable application still depends on stable probe immobilization, controlled capillary flow, low background fluorescence, probe photostability, and standardized fluorescence imaging under UV excitation.

##### Recognition-Regulated Switching

Recognition-regulated fluorescence switching and recovery systems couple fluorescence quenching with target-specific recognition or signal recovery. In these systems, fluorescence is usually quenched in an initial OFF state and then restored or further modulated after target binding or the addition of a specific recovery agent.

Zhang et al. [75] developed a µPAD using Cy5-labeled ssDNA probes and graphene oxide (GO) for Hg(II) and Ag(I) detection in food samples. Different Cy5-labeled ssDNA probes were designed for Hg(II) and Ag(I), and these probes were adsorbed onto the GO surface, where their fluorescence was quenched in the OFF state. In the presence of Hg(II) or Ag(I), the corresponding ssDNA probe specifically bound to the target ion and detached from GO, thereby restoring fluorescence to the ON state. The degree of fluorescence recovery increased with metal ion concentration, allowing quantitative determination of Hg(II) and Ag(I). Patir et al. [76] developed a µPAD using nitrogen-doped carbon dots (NCDs) for the parallel detection of Cu(II) and Hg(II). Upon contact with Cu(II) or Hg(II), the fluorescence of NCDs was quenched, corresponding to the OFF state. Trisodium citrate restored the Cu(II)-quenched fluorescence, whereas ascorbic acid restored the Hg(II)-quenched fluorescence. This ON–OFF–ON fluorescence response was used to distinguish Cu(II) and Hg(II) on the paper-based platform. Singh et al. [77] reported a µPAD using blue fluorescent carbon nanoparticles (CNPs) synthesized from waste sweet potato peels for Hg(II) detection in aqueous samples. The CNPs emitted blue fluorescence under UV light, which was quenched in the presence of Hg(II) because of the strong affinity between Hg(II) and carboxyl groups on the CNP surface. The quenched fluorescence was subsequently restored after adding ascorbic acid from fresh orange juice, which acted as a reducing agent and converted mercury species to elemental Hg(0). Fluorescence images were captured using a smartphone camera and quantitatively analyzed using a MATLAB-based algorithm based on RGB values.

Recognition-regulated fluorescence switching provides a flexible strategy for distinguishing metal ions through OFF–ON or ON–OFF–ON signal changes. Compared with direct quenching systems, these methods introduce target-specific recognition, quencher displacement, or selective fluorescence recovery. However, reliable application still depends on ssDNA or probe stability, GO or carbon-dot dispersion, stable probe immobilization on paper, controlled recovery reactions, and standardized fluorescence image analysis.

##### Ion-Imprinted QD Quenching

Ion-imprinted QD-based selective quenching combines the fluorescence response of quantum dots (QDs) with the selective recognition capability of ion-imprinted polymers (IIPs). In these systems, target ions are selectively captured by imprinted cavities near the fluorescent probes, leading to fluorescence quenching that is correlated with ion concentration. This strategy is mainly used to improve selectivity in multiplex heavy metal ion detection.

Qi et al. [78] developed a 3D origami µPAD combining ion imprinting technology with CdTe QDs for simultaneous Cu(II) and Hg(II) detection in water samples. The device contained a Y-shaped microfluidic channel and two ion-imprinted fluorescence sensing regions. CdTe QD-based IIPs served as the selective sensing elements. When Cu(II) or Hg(II) bound to the corresponding imprinted sites, fluorescence quenching occurred, and the quenching degree was used for quantitative analysis. Wang et al. [79] developed a rotary multiposition cloth–paper hybrid microfluidic device for Hg(II) and Pb(II) detection in water samples. CdTe QDs were grafted onto cotton cloth, and Hg(II)- and Pb(II)-IIPs were synthesized on the QD-modified cloth. During detection, the target ions bound to the imprinted cavities in the sensing regions, resulting in fluorescence quenching. The fluorescence intensity decrease was proportional to the concentrations of Hg(II) and Pb(II). Zhou et al. [80] reported a 3D rotating µPAD based on ZnSe QDs and ion imprinting technology for Cd(II) and Pb(II) detection in water samples. ZnSe QDs acted as fluorescent probes and emitted blue–green fluorescence under UV excitation. Imprinted cavities on the QD surface selectively recognized Cd(II) or Pb(II). After target-ion binding, interactions with amino groups on the QD surface induced fluorescence quenching, and the quenching degree was used for quantitative determination. Wang et al. [81] further developed a 3D pinwheel-shaped µPAD for simultaneous detection of Cu(II), Cd(II), Pb(II), and Hg(II) in coastal water samples. The device included a filtration area, a solid-phase extraction (SPE) unit, a detection zone, and a fluid accumulation pad. CdTe QDs were grafted onto glass fiber paper in the detection zone as fluorescent probes. Target metal ions interacted with the QDs and induced electron-transfer-related fluorescence quenching. An IIP-based SPE unit was integrated to selectively capture target ions and reduce interference from coexisting ions. Fluorescence signals were excited using a 365 nm LED, and the signal intensity was recorded after incubation.

Ion-imprinted QD-based µPADs provide a selective fluorescence-quenching format for heavy metal ion detection. In these systems, IIPs introduce target-specific recognition sites, while QDs provide measurable fluorescence responses. The integration of 3D origami, rotary, cloth–paper hybrid, and pinwheel-shaped structures further supports multiplex analysis and device integration. However, reliable application still depends on reproducible imprinting sites, long-term QD stability, uniform probe immobilization, efficient ion capture, and accurate alignment of multilayer or rotating structures.

#### 3.2.2. Fluorescence Enhancement

Fluorescence enhancement refers to an increase in fluorescence signal after target heavy metal ions interact with fluorescent probes or recognition materials. This response can be caused by metal–ligand complexation, changes in molecular conformation, restricted molecular motion, or regulation of energy transfer. According to the signal-generation mechanism, fluorescence-enhancement µPADs can be classified into ligand-complexation enhancement, nucleic-acid-mediated enhancement, and strand-displacement recovery.

##### Ligand-Complexation Enhancement

Ligand-complexation-induced fluorescence enhancement relies on the direct binding of metal ions to fluorescent ligands. This interaction changes the molecular conformation or electronic structure of the probe, resulting in enhanced fluorescence emission.

Nguyen et al. [82] introduced two distance-based µPADs for Al(III) detection in water samples. One configuration was a linear “chemometer” design, in which the length of the fluorescence band correlated with Al(III) concentration. The other was a radial design, in which the diameter of the fluorescence band was related to Al(III) concentration. A naphthalhydrazide derivative ligand was immobilized in the detection region as the fluorescent probe. When Al(III) flowed through the device, it bound to the ligand and induced fluorescence enhancement. A portable UV lamp was used as the excitation source, and the fluorescence band length or diameter served as the quantitative readout.

Ligand-complexation-induced fluorescence enhancement provides a simple turn-on strategy for visual and distance-based detection of heavy metal ions. However, reliable application still depends on ligand selectivity, probe stability, excitation conditions, and interference from competing metal ions such as Fe(III) and Cu(II).

##### Nucleic-Acid-Mediated Enhancement

Nucleic-acid-structure-mediated fluorescence enhancement uses metal-ion-triggered formation or activation of specific nucleic acid structures to generate fluorescence signals. Typical mechanisms include G-quadruplex formation, T–Hg(II)–T coordination, DNAzyme-mediated cleavage, and aptamer-regulated fluorescence recovery. In these systems, target metal ions regulate nucleic acid structures or hybridization states, thereby producing measurable fluorescence enhancement.

Sun et al. [83] designed a suspending-droplet mode µPAD (SD-µPAD) for Pb(II) detection in aqueous samples. The device used superhydrophobic patterns on an impermeable paper substrate to control droplet storage, transfer, and mixing. In this system, Pb(II) triggered the formation of an oligonucleotide G-quadruplex structure, which enhanced fluorescence after binding to an iridium complex. An LED was used as the excitation source, and the fluorescence signal was captured using a smartphone camera. Pb(II) concentration was calculated by analyzing fluorescence intensity with ImageJ. Yuan et al. [84] developed a multi-channel fluorescent µPAD for simultaneous detection of Pb(II), Hg(II), Cd(II), and As(III) in food samples. Hydrophobic channel patterns were created on filter paper by wax printing to form multiple independent detection zones. Pb(II) was detected through GR-5 DNAzyme-mediated cleavage, in which the fluorescently labeled fragment hybridized with a complementary strand to generate a signal. Hg(II) was detected based on T–Hg(II)–T coordination, where two T-rich aptamers were bridged by Hg(II) to form a luminescent duplex structure. Cd(II) and As(III) were detected through target-induced aptamer binding and fluorescence recovery. The fluorescence intensity changes were recorded using smartphone imaging and analyzed with ImageJ for quantitative detection.

These representative studies indicate that nucleic-acid-structure-mediated fluorescence enhancement provides a programmable strategy for heavy metal ion detection in µPADs. G-quadruplex structures, DNAzymes, T–Hg(II)–T coordination, and aptamer-regulated fluorescence recovery enable target-specific signal generation, while multi-channel paper designs support multiplex analysis. However, reliable application still depends on nucleic acid probe stability, controlled hybridization or cleavage efficiency, excitation-light stability, standardized smartphone imaging, and calibration in complex water and food matrices.

##### Strand-Displacement Recovery

Competitive strand-displacement fluorescence recovery is based on the release of a fluorescently labeled strand from a quenched duplex after target-ion binding. In the initial state, fluorescence is suppressed by a quencher or complementary strand. When the target ion binds to the aptamer, the fluorescent strand is released or separated from the quencher, leading to fluorescence recovery.

Yuan et al. [85] developed a µPAD for As(III) detection in water samples by combining aptamer recognition with smartphone imaging. In the reaction zone, double-stranded DNA was immobilized, consisting of a quencher-modified aptamer and a fluorescently labeled complementary strand. When As(III) was introduced, the aptamer preferentially bound to As(III), causing displacement of the fluorescent complementary strand. The released fluorescent strand was transported to the detection region by capillary action, resulting in fluorescence enhancement under excitation light. Quantitative detection of As(III) was achieved by RGB analysis of fluorescence images using ImageJ. Yan et al. [86] developed a µPAD integrating an aptamer competitive strategy with deep learning-assisted image analysis for Cd(II) detection in vegetable samples. In this system, a fluorescein amidite (FAM)- and biotin-labeled Cd(II) aptamer was hybridized with a black hole quencher (BHQ)-labeled complementary strand to form a quenched duplex, corresponding to the OFF state. The duplex was immobilized in the detection zone through biotin–streptavidin interaction. Upon Cd(II) binding, the BHQ-labeled complementary strand was displaced, thereby restoring fluorescence to the ON state. Fluorescence images were captured using a smartphone through a 520 nm filter, and a YOLOv12-based model was used for automatic localization, classification, and quantitative concentration analysis.

Competitive strand-displacement fluorescence recovery provides a programmable turn-on strategy for heavy metal ion detection in µPADs. By converting target-induced aptamer binding into fluorescence recovery, this approach enables specific signal generation and can be combined with smartphone or deep learning-assisted image analysis. However, reliable application still depends on aptamer stability, duplex hybridization efficiency, quencher–fluorophore separation, probe immobilization, and standardized image-processing models.

#### 3.2.3. Ratiometric Fluorescence

Ratiometric fluorescence detection is a sensing strategy in which analyte concentration is determined by comparing fluorescence intensity ratios at two different wavelengths. Compared with single-emission fluorescence detection, ratiometric systems provide internal signal correction because the response is based on the relative change between two emission signals rather than a single intensity value.

**Figure 7:**
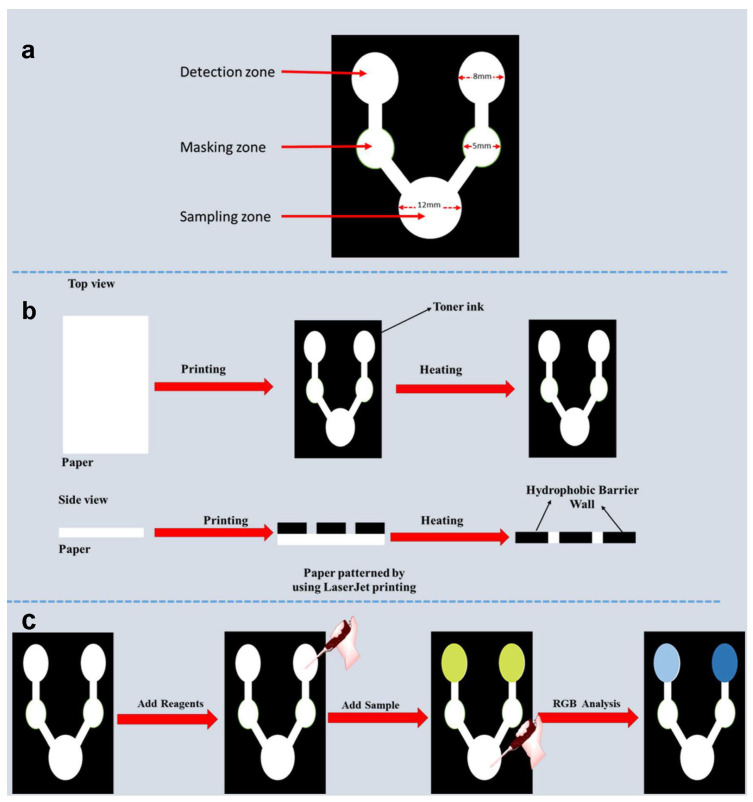
Representative µPAD for ratiometric fluorescence detection of Cu(II) and Fe(III). This figure represents a ratiometric fluorescence detection method based on a fluorescent metal–organic framework@tetracycline nanocomposite (FMOF-5@TC). (**a**) Structural design of the µPAD, including the sampling zone, masking zone, and detection zones. (**b**) Fabrication process of the µPAD by laser-jet printing and heating to form hydrophobic barriers. (**c**) Schematic workflow of reagent addition, sample introduction, fluorescence color change, and RGB analysis. The device enables simultaneous detection of Cu(II) and Fe(III) in drinking water by recording fluorescence color changes with a smartphone. Adapted with permission from Ref. [87]. Copyright 2024 The Royal Society of Chemistry.

Al-Jaf et al. [87] reported a dual-functional ratiometric µPAD for the simultaneous detection of Cu(II) and Fe(III) in drinking water (Figure 7). The device consisted of a sample area and two detection areas connected by separate channels. The first channel was used for the combined response to Cu(II) and Fe(III), whereas the second channel contained a masking area for Cu(II) detection. A fluorescent metal–organic framework (FMOF)@tetracycline (TC) nanocomposite, namely FMOF-5@TC, served as the fluorescent probe and exhibited dual-emission behavior. In the absence of Cu(II) and Fe(III), the probe emitted yellow–green fluorescence. After the addition of Cu(II) or Fe(III), complexation with the probe surface induced a shift to blue fluorescence. The fluorescence color change was recorded using a smartphone, and RGB values were analyzed to calculate the fluorescence intensity ratio for quantitative detection. Sodium fluoride (NaF) was introduced into the masking area to reduce the interference of Fe(III) during Cu(II) detection.

Ratiometric fluorescence µPADs provide a dual-emission readout format for heavy metal ion detection. By using fluorescence intensity ratios, these systems can reduce the influence of environmental or instrumental variations. However, reliable application still depends on probe stability, channel design, effective masking strategies, and control of inter-ion interference.

Collectively, fluorescence-based µPADs provide sensitive and visually distinguishable signal responses for heavy metal ion detection, but their analytical performance depends strongly on the fluorescence response mechanism, probe design, and signal acquisition mode. Fluorescence quenching is simple and easy to implement, making it suitable for rapid screening, but its selectivity can be affected by nonspecific quenching, background fluorescence, and matrix components. Fluorescence enhancement provides turn-on responses with improved visual contrast, but it requires stable fluorescent probes, controlled recognition reactions, and reliable excitation conditions. Ratiometric fluorescence offers better quantitative reliability through internal signal correction, although it generally requires more complex probe construction and ratio-based data processing. Future fluorescence-based µPADs should focus on stable fluorescent nanomaterials, selective recognition elements, low-background paper substrates, standardized smartphone-assisted imaging, and multi-signal correction strategies. Representative fluorescence-based µPADs for heavy metal ion detection are summarized in Table 3.

### 3.3. Electrochemical Detection Methods

Electrochemical detection is widely used for heavy metal ion analysis because of its high sensitivity, good quantitative capability, relatively simple instrumentation, and compatibility with portable readout systems. In µPAD-based platforms, heavy metal ions are detected by converting ion-recognition, redox, deposition, or stripping processes into measurable electrical signals, such as electrode potential or current. Compared with colorimetric and fluorescence-based methods, electrochemical µPADs are less affected by ambient light and matrix color, but their performance is more dependent on electrode fabrication, surface modification, electrode stability, calibration procedures, and fouling resistance.

**Figure 8:**
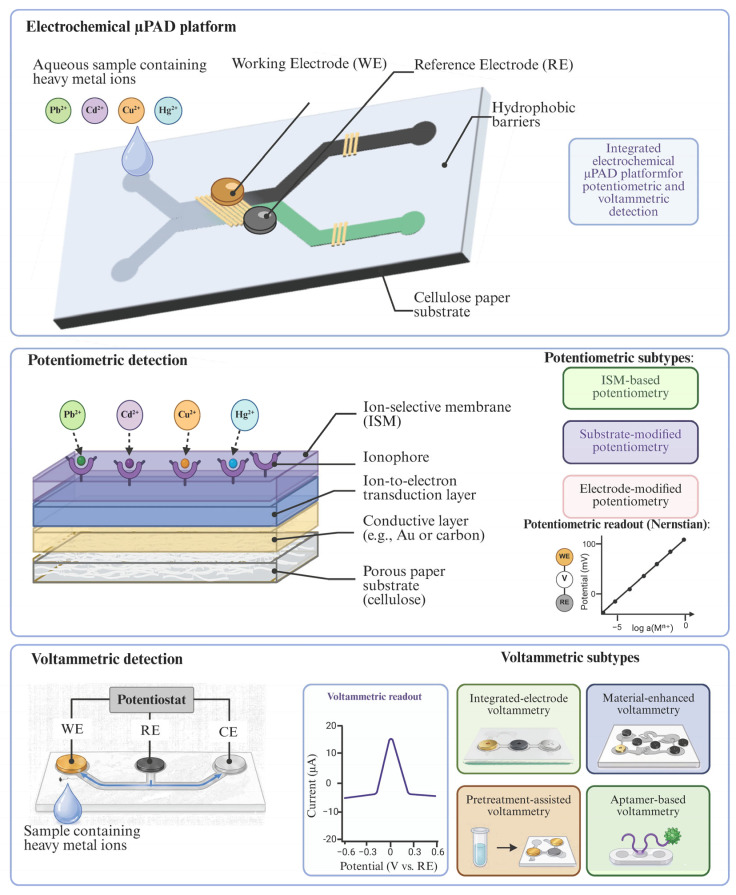
Schematic classification of electrochemical µPADs for heavy metal ion detection. Electrochemical µPADs can be broadly classified into potentiometric and voltammetric detection according to the type of electrical signal used. Potentiometric detection relies on ion-selective recognition and membrane potential generation, whereas voltammetric detection is based on metal deposition and stripping-current responses at the electrode surface. Created in BioRender. JQ, X. (2026) https://BioRender.com/cmhq467.

To reduce descriptive study-by-study presentation, this section discusses electrochemical µPADs according to signal type, electrode configuration, material modification, pretreatment integration, and recognition strategy. Based on the electrical signal used, electrochemical µPADs can be broadly classified into potentiometric detection and voltammetric detection. Potentiometric µPADs are suitable for label-free monitoring of free ion activity, whereas voltammetric µPADs, especially stripping-based systems, are more suitable for trace-level and multiplex metal detection. The classification of electrochemical µPADs for heavy metal ion detection is schematically summarized in Figure 8.

#### 3.3.1. Potentiometric Detection

Potentiometric detection measures the potential difference between a working electrode (WE) and a reference electrode (RE) under near-zero current conditions. In µPAD-based potentiometric systems, the target ion activity is converted into a potential response through ion-selective membranes, modified substrates, or modified electrode interfaces. According to the main design strategy, potentiometric µPADs can be further classified into ion-selective membrane (ISM)-based potentiometry, substrate-modified potentiometry, and electrode-modified potentiometry.

##### ISM-Based Potentiometry

ISM-based potentiometry relies on selective ion recognition within an ion-selective membrane. The target ion interacts with the ionophore in the membrane phase, causing ion redistribution across the membrane interface and generating a membrane potential. According to the Nernst equation, the potential response is related to the logarithm of target ion activity.

Ding et al. [88] designed a µPAD integrating paper-based sampling with all-solid-state Pb(II)-selective electrode detection for Pb(II) determination in wastewater samples. Cellulose filter paper pretreated with hydrochloric acid was used as the sampling medium to reduce Pb(II) adsorption on the paper surface. The Pb(II)-selective electrode functioned as the WE and generated a potential response through selective Pb(II) recognition in the membrane. The potential difference between the WE and RE showed a logarithmic relationship with free Pb(II) activity, allowing quantitative determination using a calibration curve.

Silva et al. [89] developed an ISM-modified µPAD for Pb(II) determination in environmental water samples. The sensing element of the Pb(II)-selective electrode consisted of a Pb(II)-selective membrane containing a lead ionophore, ionic additive, plasticizer, and polymer matrix. When the electrode contacted the test solution, free Pb(II) underwent reversible complexation with the ionophore within the ISM, generating a membrane potential. The potential response was then used to quantify free Pb(II) activity according to the Nernst equation.

##### Substrate-Modified Potentiometry

Substrate-modified potentiometry focuses on reducing substrate-induced errors during paper-based sampling. Because heavy metal ions can adsorb onto cellulose fibers or interact with paper components, substrate selection or chemical pretreatment is important for stabilizing the potentiometric response.

Lisak et al. [90] designed a µPAD integrating paper-based sampling with potentiometric detection for simultaneous Cd(II) and Pb(II) determination in food and environmental samples. Different paper-based sampling materials, including black ribbon ashless filter paper, hospital cotton cloth, and dust-free paper towels, were evaluated as microfluidic media. Ion-selective electrodes (ISEs) and REs were placed in direct contact with the sample-loaded substrate. The measured potential difference showed a logarithmic relationship with ion activity under constant ionic strength conditions, enabling quantitative determination using calibration curves.

Ding et al. [91] developed an inorganic salt-modified paper-based sampling strategy coupled with potentiometric detection for Cd(II) and Pb(II) measurement in water samples. Filter paper was modified by immersion in Cd(NO_3_)_2_ or Pb(NO_3_)_2_ solution, followed by washing and drying. Cd(II)- and Pb(II)-selective electrodes were then coupled with the modified paper substrate. When target ions contacted the corresponding ISEs, a membrane potential was generated, and the potential response was correlated with ion concentration according to the Nernst equation. The inorganic salt modification was used to reduce interactions between heavy metal ions and the paper substrate.

##### Electrode-Modified Potentiometry

Electrode-modified potentiometry improves the electrical properties of paper-based electrodes by introducing conductive materials or ion-to-electron transduction layers. This strategy is used to enhance potential stability, electrical conductivity, and electrode response reproducibility.

Silva et al. [92] developed a metal-modified µPAD for Pb(II) determination in environmental samples. Gold, platinum, palladium, and other metals were deposited onto the paper substrate by sputtering to form conductive metal layers. The Pb(II)-selective electrode was constructed using a Pb(II)-selective PVC membrane, a conductive poly(3,4-ethylenedioxythiophene):poly(styrenesulfonate) (PEDOT:PSS) ion-to-electron transduction layer, and an internal Ag/AgCl RE. When the electrode contacted the sample solution, Pb(II) interacted with the selective membrane, producing ion redistribution across the membrane interface and generating a potential response. The potential difference was used for quantitative Pb(II) determination based on the logarithmic relationship with free Pb(II) activity.

Potentiometric µPADs provide a label-free strategy for detecting free heavy metal ion activity through electrode potential responses. ISM-based systems provide ion-selective recognition, substrate-modified systems reduce paper-induced adsorption and sampling errors, and electrode-modified systems improve conductivity and ion-to-electron transduction. However, reliable application still depends on membrane selectivity, substrate pretreatment reproducibility, electrode stability, and robust calibration in complex samples.

#### 3.3.2. Voltammetric Detection

Voltammetric detection is an electrochemical method in which the electrode potential is controlled and the resulting current response is recorded. In µPAD-based heavy metal ion detection, voltammetric methods usually rely on metal deposition, preconcentration, and subsequent stripping at the working electrode surface. According to the main design strategy, voltammetric µPADs can be classified into integrated-electrode voltammetry, material-enhanced voltammetry, pretreatment-assisted voltammetry, and aptamer-based voltammetry.

##### Integrated-Electrode Voltammetry

Integrated-electrode voltammetry focuses on incorporating complete three-electrode systems into paper-based microfluidic platforms. In these devices, the WE, RE, and counter electrode (CE) are fabricated by printing, lamination, sputtering, or direct integration with paper channels, enabling sample transport and electrochemical detection within a compact format.

Yu et al. [93] developed a laminated polycaprolactone/paper/silver electrode (LPSE) for Pb(II) detection in water samples. Filter paper served as the substrate, and silver paste was used to form the WE, CE, and RE. Polycaprolactone (PCL) was used as the insulating and encapsulating material. Square-wave anodic stripping voltammetry (SWASV) was employed for detection. During the deposition step, Pb(II) was reduced and deposited onto the WE surface. The deposited lead was then reoxidized during the anodic stripping step, generating a stripping peak current proportional to Pb(II) concentration. Shen et al. [94] developed an unmodified graphite foil-based paper microfluidic device for Cd(II) and Pb(II) detection in water samples. The device used a three-electrode system composed of graphite foil, with the WE and CE arranged vertically and separated by a microfluidic paper channel. A quasi-reference electrode was positioned near the WE. SWASV was used for detection. Cd(II) and Pb(II) were first reduced and deposited on the graphite WE surface, followed by anodic stripping to generate characteristic peak currents for quantitative analysis. Kokkinos et al. [95] developed an ePAD for simultaneous Cd(II) and Zn(II) detection in environmental samples. Wax-printed paper channels were combined with a sputtered thin-film electrode system consisting of a Sn-film WE, Ag RE, and Pt CE. During SWASV detection, Cd(II) and Zn(II) were reduced and preconcentrated on the Sn-film WE, followed by reoxidation during the positive potential scan. The stripping peak currents were used for quantitative determination. Smith et al. [96] designed an electrochemical µPAD integrating wax-patterned paper channels with screen-printed electrodes for Pb(II) and Cd(II) detection in water samples. Ag/AgCl ink was used to form the RE and conductive tracks, while carbon ink was used to construct the WE and CE. SWASV was applied for detection. Pb(II) and Cd(II) were electrodeposited on the WE during the deposition step and then oxidatively stripped at characteristic potentials, allowing qualitative identification by peak potential and quantitative analysis by peak current.

Integrated-electrode voltammetry µPADs simplify device operation by combining microfluidic sampling and electrochemical detection in one platform. Their performance mainly depends on electrode fabrication reproducibility, printed or sputtered electrode stability, uniform sample transport, and reliable stripping peak generation.

##### Material-Enhanced Voltammetry

Material-enhanced voltammetry improves electrochemical response by modifying the WE with nanomaterials, conductive carbon materials, metal films, or other functional electrode materials. These modifications can increase active surface area, promote metal deposition, enhance electron transfer, and improve stripping current response.

Pungjunun et al. [97] developed a multi-step paper-based analytical device integrated with AuNP-modified boron-doped diamond (BDD) electrodes for total inorganic arsenic detection in rice samples. The device contained modification, WE, and detection regions. AuNPs were electrodeposited onto the BDD electrode surface from Au(III) solution. As(III) was detected directly, whereas total inorganic arsenic required prior reduction of As(V) to As(III). During SWASV detection, As(III) was reduced and deposited as As(0) on the AuNP-modified BDD electrode and then reoxidized during the anodic scan to generate a stripping peak. Nantaphol et al. [98] designed a µPAD incorporating a boron-doped diamond paste electrode (BDDPE) for Cd(II) and Pb(II) detection in drinking water. The BDDPE was prepared by mixing BDD powder with mineral oil and was used as the WE. A bismuth-film-modified BDDPE was introduced to enhance the stripping response. During square-wave voltammetry (SWV), Cd(II) and Pb(II) were reduced and preconcentrated on the modified WE, followed by anodic stripping to generate characteristic peak currents. Wei et al. [99] developed an origami electrochemical µPAD integrated with nitrogen-doped graphene (NG) for simultaneous Cd(II), Pb(II), and Hg(II) detection in water samples. The device contained two paper layers, with the CE and RE on one layer and the WE on the other. NG was synthesized in situ on a paper-based screen-printed carbon electrode. During detection, heavy metal ions were attracted to nitrogen sites on the NG surface and reduced to metallic states. Differential pulse voltammetry (DPV) was then used to strip and detect the deposited metals.

Material-enhanced voltammetry µPADs improve detection performance by promoting metal enrichment, electron transfer, and stripping signal generation. However, reliable application still depends on reproducible material modification, stable film or nanomaterial formation, electrode surface uniformity, and batch-to-batch consistency.

##### Pretreatment-Assisted Voltammetry

Pretreatment-assisted voltammetry integrates sample cleanup, interference elimination, selective separation, or analyte enrichment before electrochemical detection. This strategy is useful for complex biological, food, environmental, and aerosol samples, where matrix components or coexisting ions may affect voltammetric signals.

**Figure 9:**
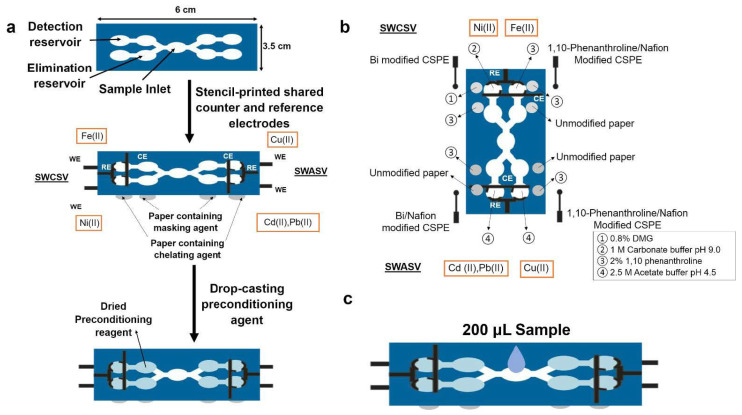
Representative Janus electrochemical µPAD for multiplex voltammetric detection of metal ions in aerosol samples. This figure represents electrochemical stripping voltammetric detection strategies in µPADs, including SWASV for Cd(II), Pb(II), and Cu(II), and SWCSV for Fe(II) and Ni(II). (**a**) Design and fabrication workflow of the Janus ePAD, including detection reservoirs, elimination reservoirs, sample inlet, stencil-printed shared CE and RE, independent WEs, and paper regions containing masking or chelating agents. (**b**) Layout of the Janus ePAD showing different modified carbon screen-printed electrodes (CSPEs) and reagent zones for metal-specific detection. (**c**) Sample introduction into the device for multiplex electrochemical analysis. Adapted with permission from Ref. [102]. Copyright 2019 American Chemical Society.

Wang et al. [100] developed an electrochemical µPAD for Pb(II) detection in urine samples. The device combined patterned filter paper with a detachable three-electrode system consisting of a gold-plated plastic WE, platinum wire CE, and Ag/AgCl RE. The sample was transported through paper microchannels into the detection area. Anodic stripping voltammetry (ASV) was used for Pb(II) detection, in which Pb(II) was first reduced and accumulated on the WE surface and then oxidized during anodic scanning to produce a stripping peak. Ammonium sulfate-modified filter paper was used for in situ removal of protein interference during sample transport. Hu et al. [101] developed a multifunctional µPAD for Cd(II) detection in water samples. The device consisted of a sample filtration area, an ion-imprinted polymers-modified paper (IIPs@paper) separation area, an electrochemical detection area, and a waste collection area. Cd(II) was selectively captured in the IIPs@paper region and transported to the detection area. The electrode surface was modified with reduced graphene oxide (rGO). Cd(II) was reduced and deposited on the rGO-modified paper-based screen-printed carbon electrode, followed by stripping oxidation to generate a current response proportional to Cd(II) concentration. Mettakoonpitak et al. [102] developed an electrochemical µPAD for Cd(II), Pb(II), Cu(II), Fe(II), and Ni(II) detection in aerosol samples (Figure 9). The device contained a sample inlet zone, an interference elimination zone, and a detection zone. Each channel contained an independent WE, while the RE and CE were shared. SWASV was used for Cd(II), Pb(II), and Cu(II), in which metal ions were reduced and deposited on a bismuth/Nafion-modified electrode and then anodically stripped. Square-wave cathodic stripping voltammetry (SWCSV) was used for Fe(II) and Ni(II), where metal–ligand complexes accumulated on the electrode and were reduced during the cathodic scan. On-chip pretreatment with masking or complexing reagents was used to reduce interference.

Pretreatment-assisted voltammetric µPADs improve the applicability of electrochemical detection in complex matrices by integrating matrix cleanup, selective capture, enrichment, or interference elimination. Their reliability still depends on pretreatment efficiency, reagent compatibility, stable electrode modification, controlled fluid transport, and minimized cross-interference among metal ions.

##### Aptamer-Based Voltammetry

Aptamer-based voltammetry introduces target-specific nucleic acid recognition into electrochemical µPADs. In many systems, a labeled aptamer is initially immobilized on the electrode surface through hybridization with a complementary strand. After target binding, the aptamer dissociates from the electrode, causing a decrease in electrochemical signal. This signal-off response is then used for quantitative detection.

Qian et al. [103] developed an aptamer-based electrochemical µPAD for simultaneous Cd(II) and Pb(II) detection in fruit and vegetable samples. The three-electrode system was fabricated on filter paper by screen printing. AuNPs were modified on the WE surface, and complementary DNA sequences were immobilized through Au–S bonds. Cd(II)- and Pb(II)-specific aptamers labeled with methylene blue (MB) and ferrocene (Fc), respectively, hybridized with the complementary strands to form duplex structures. In the presence of Cd(II) or Pb(II), target binding induced aptamer dissociation from the electrode surface, resulting in decreased electrochemical signals from MB or Fc. The signal decrease was used for quantitative analysis. Yuan et al. [104] constructed a multi-channel aptamer-based electrochemical µPAD for Pb(II), Cd(II), and Hg(II) detection in crab meat samples. The device contained a central injection zone and independent detection zones for different metal ions. Each detection zone integrated a screen-printed three-electrode system. A complementary probe was immobilized on the electrode surface, and an MB-labeled aptamer was hybridized with it to form an aptamer–complementary probe duplex. When the target ion was present, it preferentially bound to the aptamer, causing release of the MB-labeled aptamer from the electrode surface. Square-wave voltammetry (SWV) was used to monitor the decrease in electrochemical signal, which was proportional to target ion concentration.

Aptamer-based voltammetric µPADs combine electrochemical signal transduction with target-specific nucleic acid recognition. They are suitable for multiplex detection because different aptamers and electrochemical reporters can be assigned to different targets or channels. However, reliable application still depends on aptamer stability, DNA immobilization efficiency, reporter signal separation, detection-zone isolation, and control of matrix effects in complex food samples.

Taken together, electrochemical µPADs provide quantitative and portable strategies for heavy metal ion detection by converting ion-recognition or redox processes into measurable potential or current signals. Potentiometric detection is simple, low-power, and suitable for monitoring free metal ion activity, but its reliability depends strongly on ion-selective membrane performance, reference electrode stability, substrate pretreatment, and calibration in complex samples. Voltammetric detection generally provides higher sensitivity and better multiplexing capability, especially when stripping voltammetry is combined with integrated electrodes, nanomaterial modification, on-chip pretreatment, or aptamer recognition. However, voltammetric µPADs require reproducible electrode fabrication, controlled deposition conditions, stable surface modification, and effective management of electrode fouling and matrix interference. Future development should therefore focus on standardized electrode manufacturing, stable anti-fouling materials, integrated sample pretreatment, miniaturized electrochemical readers, and validation in real environmental, food, biological, and aerosol samples. Representative electrochemical µPADs for heavy metal ion detection are summarized in Table 4.

### 3.4. Dual-Detection Systems and Emerging Methods

**Figure 10:**
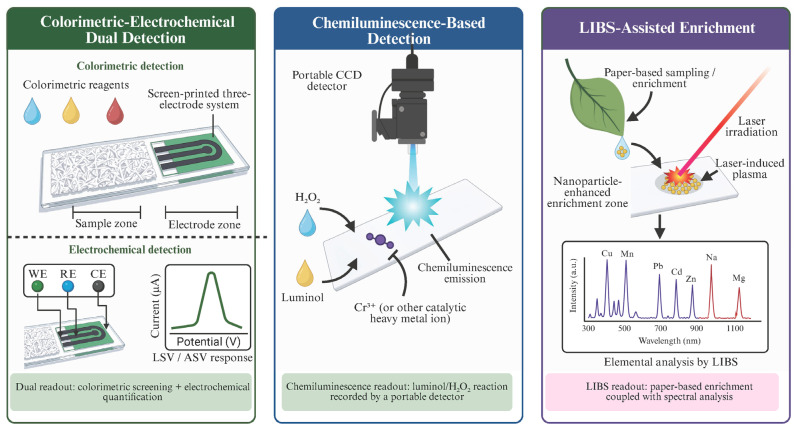
Schematic classification of dual-detection systems and emerging µPAD-based methods for heavy metal ion detection. Colorimetric–electrochemical dual detection combines visual colorimetric readout with electrochemical current signals, enabling complementary screening and quantitative analysis. Chemiluminescence-based detection relies on light emission generated by metal-catalyzed luminol/H_2_O_2_ reactions and recorded using a portable optical detector. LIBS-assisted enrichment integrates paper-based sampling or preconcentration with laser-induced breakdown spectroscopy for elemental spectral readout. The arrows indicate sample transport, reaction, signal generation, and detection/readout processes. The different colored panels represent different detection strategies, including colorimetric–electrochemical dual detection, chemiluminescence-based detection, and LIBS-assisted enrichment. These strategies expand the analytical scope of conventional single-mode µPADs by introducing complementary signal modes or advanced instrumental readout. Created in BioRender. JQ, X. (2026) https://BioRender.com/k8x4cb9.

Beyond single-mode colorimetric, fluorescence, and electrochemical detection, several µPAD-based systems have been developed by integrating complementary signal readout strategies or emerging analytical techniques. These approaches can expand the detectable target range, improve signal confirmation, and support field-oriented heavy metal ion analysis. According to the signal strategy and system configuration, these methods can be classified into colorimetric–electrochemical dual detection, chemiluminescence-based detection, and laser-induced breakdown spectroscopy (LIBS)-assisted enrichment platforms. Representative dual-detection systems and emerging µPAD-based methods for heavy metal ion analysis are schematically summarized in Figure 10.

#### 3.4.1. Colorimetric–Electrochemical Dual Detection

Colorimetric–electrochemical dual detection combines the visual readability of colorimetric assays with the quantitative capability of electrochemical detection. In these systems, colorimetric readout is generally used for rapid screening or selected ion detection, whereas electrochemical readout provides current-based quantitative analysis.

An et al. [105] developed a µPAD for simultaneous determination of total Cr and Cr(VI) in water samples. In the colorimetric module, Cr(III) was first oxidized to Cr(VI) by preloaded Ce(IV), and Cr(VI) then reacted with 1,5-DPC to form a purple complex. Smartphone images of the colorimetric region were analyzed using ImageJ for total Cr determination. In the electrochemical module, linear sweep voltammetry (LSV) was used to detect Cr(VI), where Cr(VI) was reduced to Cr(III) under acidic conditions and generated a reduction peak current proportional to Cr(VI) concentration. Chaiyo et al. [106] developed a dual-detection µPAD for Pb(II), Cd(II), and Cu(II) analysis in environmental and food samples. In the colorimetric region, Cu(II) catalyzed thiosulfate-induced etching of silver nanoplatelets, resulting in a color change from pink–purple to nearly colorless. The color response was evaluated visually or by digital image analysis. In the electrochemical region, Pb(II) and Cd(II) were detected by ASV using a bismuth-modified boron-doped diamond electrode. During measurement, Pb(II) and Cd(II) were preconcentrated on the electrode surface and then reoxidized to generate stripping peak currents. Hermansyah et al. [107] reported a µPAD for Cd(II), Pb(II), and Cu(II) detection in mineral water. Cu(II) was detected colorimetrically using functionalized AuNPs. In the presence of Cu(II), ligand-mediated AuNP aggregation caused a surface plasmon resonance shift and a visible color change from pink to blue. The color intensity was measured using an RGB sensor. Cd(II) and Pb(II) were detected by SWASV after bismuth film deposition on the working electrode, where the target ions were reduced, accumulated, and then anodically stripped to generate characteristic current peaks.

Silva-Neto et al. [108] developed a plug-and-play platform combining a colorimetric µPAD with an electrochemical paper-based analytical device for six metal ions in river water. Fe(II), Ni(II), and Cu(II) were detected colorimetrically through reactions with 1,10-phenanthroline, dimethylethylenediamine, and pyridinoxazole, respectively. Zn(II), Cd(II), and Pb(II) were detected using SWASV on the electrochemical module, where the metals were deposited on the modified working electrode and then stripped during the positive potential scan.

Colorimetric–electrochemical dual-detection systems broaden µPAD-based analysis by combining visual screening with electrochemical quantification. However, reliable application still depends on the compatibility of colorimetric reagents and electrochemical components, stable electrode modification, coordinated sample flow, and effective integration of two signal readout modes.

#### 3.4.2. Chemiluminescence-Based Detection

Chemiluminescence-based µPADs detect heavy metal ions by measuring light emission generated from metal-catalyzed or metal-regulated chemiluminescent reactions. The luminol–H_2_O_2_ system is commonly used, in which catalytic metal ions can promote luminol oxidation and generate a measurable chemiluminescent signal.

Alahmad et al. [109] reported a wax-printed µPAD for Cr(III) detection in aqueous samples. The device contained six independent channels, each consisting of a reagent injection zone, a reaction zone, and a waste zone. Cr(III) catalyzed the luminol–H_2_O_2_ reaction to generate chemiluminescence. The emitted light was collected through optical fibers and transmitted to a photomultiplier tube (PMT), and the light intensity was used for quantitative Cr(III) analysis. Shang et al. [110] proposed a gravity- and capillary-driven chemiluminescence flow µPAD for Cr(III) detection in water samples. The device consisted of a loading zone, flow channel, and detection zone, with liquid flow driven by gravity and capillary force. Cr(III) catalyzed the oxidation of luminol by H_2_O_2_, producing chemiluminescence. The emitted signal was recorded using a portable charge-coupled device (CCD) camera, and Cr(III) concentration was determined from the chemiluminescence intensity.

Chemiluminescence-based µPADs provide an optical detection format with low background because no external excitation light is required. However, their performance depends on luminol and H_2_O_2_ stability, pH and reaction-time control, efficient reagent mixing, ambient-light protection, and portable signal acquisition.

#### 3.4.3. LIBS-Assisted Enrichment Platforms

LIBS-assisted enrichment platforms integrate paper-based sampling or preconcentration with laser-induced breakdown spectroscopy (LIBS). In these systems, the paper-based device mainly serves as a sampling and enrichment platform, whereas LIBS provides elemental readout for quantitative analysis.

Yang et al. [111] developed a wearable paper-based microfluidic sap enrichment device combined with LIBS for Pb(II) and Cd(II) detection in cucumber plants. The device was inserted into the plant stem, where sap containing heavy metal ions was transported by capillary action to an enrichment filter paper. Colorimetric detection was first used to optimize enrichment conditions, with xylenol orange for Pb(II) and dithizone for Cd(II). Smartphone images were analyzed by extracting RGB values. For LIBS analysis, the enrichment filter paper was modified with copper nanoparticles to enhance heavy metal adsorption and amplify LIBS signals. The enriched filter paper was then analyzed by LIBS for elemental quantification.

LIBS-assisted enrichment platforms extend µPAD applications from liquid samples to in vivo or minimally invasive plant analysis. This strategy links passive paper-based sampling, nanoparticle-assisted enrichment, and instrumental elemental readout. However, practical application still depends on stable sap transport, reproducible enrichment, nanoparticle modification stability, and the availability of portable LIBS instrumentation.

From a comparative perspective, dual-detection systems and emerging µPAD-based methods expand the analytical scope of conventional paper-based platforms by integrating complementary signal modes or advanced instrumental readout. Colorimetric–electrochemical dual detection combines rapid visual screening with current-based quantification, but its performance depends on the compatibility between colorimetric reagents and electrochemical components. Chemiluminescence-based detection provides low-background light-emission readout without external excitation, but it requires strict control of reagent stability, reaction time, and ambient-light interference. LIBS-assisted enrichment platforms link paper-based sampling with elemental analysis and are useful for complex or in vivo samples, but they usually require auxiliary instrumentation. Therefore, future development should focus on improving signal compatibility, simplifying device operation, enhancing reagent and electrode stability, and integrating portable readout systems. Representative dual-detection systems and emerging µPAD-based methods for heavy metal ion detection are summarized in Table 5.

**Figure 11:**
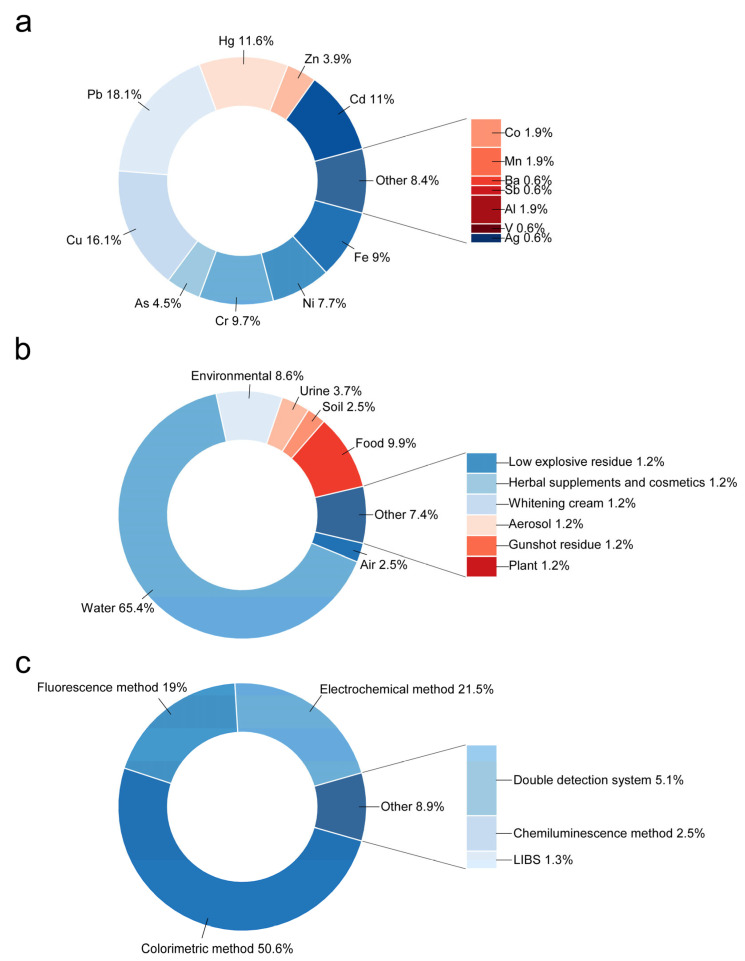
Statistical analysis of reported µPADs for heavy metal ion detection based on the literature summarized in this review. This figure represents a literature-based overview of target heavy metal ions, sample matrices, and detection methods used in reported µPAD studies. (**a**) Distribution of target heavy metal ions. (**b**) Distribution of sample matrices. (**c**) Distribution of detection methods. Percentages were calculated from the original counts and rounded to one decimal place; therefore, the sums of rounded subcategories may differ slightly from the corresponding “Other” percentages.

Taken together, the literature summarized in this review shows clear trends in the development of paper-based microfluidic devices for heavy metal ion detection. As shown in Figure 11a, Pb and Cu are the most frequently investigated target metals, accounting for 18.1% and 16.1% of the reported studies, respectively, followed by Hg, Cd, Cr, Fe, Ni, As, and Zn. This distribution indicates that current µPAD-based studies mainly focus on highly toxic and widely regulated heavy metals related to environmental pollution, food safety, and public health. In terms of sample matrices, water samples represent the dominant application scenario, accounting for 65.4% of the reported studies, followed by food and environmental samples (Figure 11b). This suggests that water quality monitoring remains the primary application field of µPADs, whereas applications in more complex matrices, such as food, soil, air, urine, cosmetics, plants, and residues, still require further development. Regarding detection methods, colorimetric detection is the most widely used strategy, accounting for 50.6% of the reported methods, followed by electrochemical methods and fluorescence methods (Figure 11c). These results further demonstrate that the field is currently dominated by colorimetric µPADs and aqueous sample analysis, while future development is expected to move toward multiplexed detection, complex matrix analysis, improved anti-interference capability, and more accurate quantitative readout strategies.

## 4. Critical Comparison and Future Perspectives

Several previous reviews have provided important foundations for paper-based and microfluidic platforms for heavy metal ion detection. Lin et al. reviewed paper-based microfluidics for heavy metal detection and discussed paper substrates, fabrication methods, capillary-driven flow, and major detection mechanisms, including colorimetric, fluorescence-based, and electrochemical detection [112]. Meredith et al. summarized the development and application of paper-based analytical devices for environmental analysis [113], while Fu and Wang reviewed general detection methods and applications of µPADs, including materials, fabrication methods, driving mechanisms, and analytical readout strategies [114]. In addition, Mesquita et al. discussed low-cost microfluidic systems for environmental monitoring [115], and Filippidou and Chatzandroulis reviewed broader microfluidic and lab-on-a-chip platforms for heavy metal ion detection [13]. These reviews have greatly advanced the understanding of paper-based and microfluidic sensing technologies. However, many of them focused on earlier developments, broader environmental applications, general µPAD technologies, low-cost microfluidic systems, or microfluidic platforms not limited to paper-based devices.

Compared with these previous reviews, the present review provides an updated and more focused contribution by concentrating specifically on µPAD-based heavy metal ion detection reported from 2015 to 2025. In addition to summarizing recent studies, this review reorganizes the field from the perspectives of device concepts, material systems, sensing mechanisms, signal readout strategies, sample matrices, and practical challenges. Furthermore, colorimetric, fluorescence-based, electrochemical, dual-detection, and emerging methods are further classified into specific subcategories according to their signal-generation principles and device configurations. Therefore, this review aims not only to update the literature but also to provide a structured and critical framework for understanding the current status, remaining challenges, and future development of µPADs for heavy metal ion detection.

Based on this positioning, the following discussion further compares the major µPAD-based detection strategies from a critical perspective. Although µPAD-based platforms have been widely explored for heavy metal ion detection, their analytical performance and practical applicability are strongly dependent on the sensing mechanism, device configuration, signal readout mode, and sample matrix. Therefore, beyond summarizing individual studies, it is necessary to compare the major detection strategies from the perspectives of sensitivity, operational simplicity, quantitative capability, multiplexing potential, and field applicability.

Colorimetric µPADs remain the most widely used format because they are low-cost, easy to fabricate, and compatible with naked-eye observation or smartphone-assisted image analysis. These advantages make them suitable for rapid screening and resource-limited settings. However, colorimetric signals are easily affected by illumination conditions, paper background, reagent distribution, color uniformity, and matrix color. Therefore, although colorimetric µPADs are attractive for on-site qualitative or semi-quantitative analysis, their quantitative reliability still requires standardized imaging conditions, internal calibration, and improved anti-interference strategies.

Fluorescence-based µPADs generally provide higher sensitivity and better signal contrast than simple colorimetric systems. Fluorescence quenching, fluorescence enhancement, and ratiometric fluorescence strategies allow more flexible signal regulation and can improve detection sensitivity for trace metal ions. In particular, ratiometric systems can partially correct environmental and instrumental fluctuations by using two emission signals. Nevertheless, fluorescence-based µPADs usually require external excitation sources, optical filters, or fluorescence imaging devices, which may increase system complexity. In addition, probe photostability, background fluorescence from paper substrates, and fluorescence quenching by coexisting substances remain important challenges for real-sample analysis.

Electrochemical µPADs show strong potential for quantitative and sensitive heavy metal ion detection because electrical signals are less dependent on ambient light and can be directly recorded using portable electrochemical workstations. Potentiometric µPADs are suitable for ion-selective and label-free sensing, whereas voltammetric µPADs are particularly useful for trace-level detection through deposition and stripping processes. However, the performance of electrochemical µPADs is closely related to electrode fabrication, surface modification, reference electrode stability, ion-to-electron transduction, and electrode fouling. Therefore, reproducible electrode preparation, stable paper-based reference electrodes, and robust calibration in complex matrices are essential for practical applications.

Dual-detection systems and emerging µPAD-based methods provide additional analytical reliability by integrating complementary signal modes, such as colorimetric–electrochemical readout, chemiluminescence, or LIBS-assisted elemental analysis. These strategies can improve confirmation capability, reduce false-positive results, or expand the detectable range of heavy metal ions. However, compared with single-mode µPADs, they often require more complex device structures, additional reagents, or auxiliary instruments. The key challenge is therefore to balance analytical performance with simplicity, portability, and cost-effectiveness.

From the perspective of sample matrices, most reported µPADs have been validated in water samples, which are relatively easy to handle and compatible with direct paper-based analysis. In contrast, food, biological, soil, plant, and aerosol samples usually contain more complex matrix components and often require extraction, filtration, dilution, digestion, or preconcentration steps. These pretreatment procedures may improve sensitivity and selectivity but can also weaken the simplicity of µPAD-based assays. Future µPAD development should therefore place greater emphasis on integrated sample pretreatment, matrix-tolerant sensing chemistries, and validation using real samples rather than only spiked model samples.

Overall, the future development of µPADs for heavy metal ion detection should focus on several directions. First, device design should move from simple proof-of-concept formats toward reproducible and manufacturable platforms. Second, sensing materials and reagents should be optimized for long-term storage stability and reduced batch-to-batch variation. Third, signal acquisition should be standardized through internal references, smartphone-based calibration, or machine-learning-assisted image analysis. Fourth, multiplexed detection should be improved to meet the need for simultaneous monitoring of multiple metal ions in environmental and food samples. Finally, more attention should be paid to field validation, inter-laboratory comparison, and practical performance under real operating conditions. These improvements will be essential for translating µPADs from laboratory prototypes into reliable tools for on-site heavy metal ion monitoring.

## 5. Conclusions

Over the past decade (2015–2025), µPADs have developed into promising platforms for rapid, low-cost, and on-site detection of heavy metal ions. By integrating capillary-driven fluid transport, flexible paper substrates, functional sensing materials, and diverse signal readout strategies, µPADs provide practical alternatives to conventional laboratory-based techniques that require expensive instruments, skilled operators, and complex sample preparation. Recent advances in colorimetric, fluorescence-based, electrochemical, dual-detection, and emerging sensing strategies have expanded µPAD applications from simple qualitative screening to more sensitive, quantitative, and multiplexed analysis in environmental, food, biological, and other complex samples.

Despite these advances, challenges related to device reproducibility, reagent stability, matrix interference, signal standardization, and real-sample validation still limit broader practical application. With continued progress in materials engineering, device integration, portable readout systems, and intelligent data analysis, µPADs are expected to become increasingly important tools for heavy metal ion monitoring, especially in environmental surveillance, food safety, public health protection, and resource-limited field analysis.

## Figures and Tables

**Table 1:** Detection parameters of the direct colorimetric method for heavy metal ion detection.

Heavy Metal Ion	Reported LOD	Linear Detection Range	Sample	Chromogenic Reagent	Readout	Reference
Al(III)	0.025 µg	-	Low-explosive residue	Ammonium acetate and aluminon	Visual colorimetric screening	[40]
	0.025 µg	-	Low explosive residue	5-Br-PAPS	Distance-based visual readout	[46]
V(IV)	4,000,000 µgL^−1^	4,000,000–20,000,000 µgL^−1^	Herbal supplements and cosmetics	5-Br-PAPS	Distance-based visual readout	[46]
Cr(III)	80 μgL^−1^	100–60,000 μgL^−1^	Water	DPC	Dual-channel colorimetric analysis	[54]
Cr(VI)	8 μgL^−1^	20–100,000 μgL^−1^	Water	DPC	Dual-channel colorimetric analysis	[54]
	30,000 μgL^−1^	40,000–400,000 μgL^−1^	Water	1,5-DPC	RGB analysis	[35]
	-	0–10,000 μgL^−1^	Water	1,5-DPC	ImageJ-based imaging analysis	[61]
	1000 μgL^−1^	50,000–250,000 μgL^−1^	Industrial wastewater	DPC	ImageJ analysis	[63]
	1000 μgL^−1^	250,000–600,000 μgL^−1^	Industrial wastewater	DPC	ImageJ analysis	[63]
	500 µgL^−1^	50–10,000 µgL^−1^	Water	1,5-DPC	Visual colorimetric screening	[56]
	180 µgL^−1^	30–20,000 µgL^−1^	Water	1,5-DPC	ImageJ-based RGB analysis	[57]
	180 µgL^−1^	500–10,000 µgL^−1^	Water	1,5-DPC	Visual colorimetric analysis	[59]
	0.152 µg	0.15–3.810 µg	Air	Selective colorimetric reagents	Smartphone-based grayscale analysis	[66]
Total Cr	250 μgL^−1^	25–100,000 µgL^−1^	Environment	1,5-DPC	Smartphone image analysis	[41]
Mn(II)	110 µgL^−1^	11–550 µgL^−1^	Water	PAR	ImageJ-based image analysis	[58]
	890 µgL^−1^	-	Water	Commercial test strips	Colorimetric screening followed by LIBS analysis	[67]
	0.01008 µg	0.01008–1.008 µg	Air	Selective colorimetric reagents	Smartphone-based grayscale analysis	[66]
Fe(II)	0.186 µg	0.186–1.86 µg	Air	Selective colorimetric reagents	Smartphone-based grayscale analysis	[66]
	0.0166 µg	0.0166–0.8285 µg	Air	1,10-Phenanthroline	Smartphone image analysis	[65]
	36 µgL^−1^	50–800 µgL^−1^	Water	BSP-SP	Smartphone-based grayscale analysis	[39]
Fe(III)	3960 µM	8.9–89 µM	Water	Phenanthroline	Absorbance	[34]
	40,000 µgL^−1^	40,000–350,000 µgL^−1^	Water	1,10-Phenanthroline	Image-based analysis	[36]
	100 µM	100–5000 µM	Water	1,10-Phenanthroline	ImageJ-based grayscale analysis	[55]
	1100 µgL^−1^	500–15,000 µgL^−1^	Water	Bathophenanthroline	Smartphone-based grayscale analysis	[24]
	300 μgL^−1^	100–20,000 μgL^−1^	Drinking water	Bathophenanthroline	Smartphone-based digital image colorimetry	[60]
	0.25 µg	-	Low explosive residue	paminophenol	Visual colorimetric screening	[40]
	2,000,000 µgL^−1^	2,000,000–10,000,000 µgL^−1^	Herbal supplements and cosmetics	5-Br-PAPS	Distance-based visual readout	[46]
Co(II)	590 µgL^−1^	590–4710 µgL^−1^	Water	PAR	ImageJ-based image analysis	[58]
	2,000,000 µgL^−1^	2,000,000–10,000,000 µgL^−1^	Herbal supplements and cosmetics	5-Br-PAPS	Distance-based visual readout	[46]
	0.00816 µg	0.00816–0.0816 µg	Air	Selective colorimetric reagents	Smartphone-based grayscale analysis	[66]
Ni(II)	500 µgL^−1^	500–10,000 µgL^−1^	Water	DMG	Visual colorimetric analysis	[56]
	500 µM	1000–50,000 µM	Water	DMG	Smartphone-based grayscale analysis	[55]
	240 µgL^−1^	30–20,000 µgL^−1^	Water	DMG	ImageJ-based RGB analysis	[57]
	4800 µgL^−1^	15,000–60,000 µgL^−1^	Water	DMG	Visual colorimetric analysis	[59]
	2000 µgL^−1^	1000–50,000 µgL^−1^	Water	DMG	Smartphone-based grayscale analysis	[24]
	5870 µgL^−1^	5870–352,160 µgL^−1^	Water	DMG	ImageJ-based image analysis	[58]
	1300 μgL^−1^	1000–20,000 μgL^−1^	Drinking water	DMG	Smartphone-based digital image colorimetry	[60]
	0.0099 µg	0.0099–0.4964 µg	Air	DMG	Smartphone image analysis	[65]
	0.0804 µg	0.0804–0.804 µg	Air	Selective colorimetric reagents	Smartphone-based grayscale analysis	[47]
Cu(II)	0.340 µM	1–7 µM	Water	PAN	Euclidean distance method	[37]
	1700 µgL^−1^	5000–1,400,000 µgL^−1^	Water	Chrome azurol S	ImageJ-based RGB analysis	[53]
	1900 µgL^−1^	5000–200,000 µgL^−1^	Water	Catechol violet	ImageJ-based RGB analysis	[53]
	1000 μgL^−1^	1000–100,000 μgL^−1^	Soil	Sodium diethyldithiocarbamate (DDTC)	Distance-based and ImageJ-based grayscale analysis	[45]
	27.1 µgL^−1^	100–10,000 µgL^−1^	Urine	PAN	Digital image analysis	[38]
	20 µgL^−1^	20–1000 μgL^−1^	Water	CysA@AuNPs	UV-Vis analysis	[48]
	800 µgL^−1^	800–10,000 µgL^−1^	Water	Bathocuproine	Visual colorimetric screening	[56]
	1600 µgL^−1^	5000–80,000 µgL^−1^	Water	Bathocuproine	Visual colorimetric analysis	[59]
	300 µgL^−1^	100–5000 µgL^−1^	Water	Bathocuproine	Smartphone-based grayscale analysis	[24]
	320 µgL^−1^	320–63,550 µgL^−1^	Water	Bathocuproine	ImageJ-based image analysis	[58]
	924 µgL^−1^	-	Water	Commercial test strips	Colorimetric screening followed by LIBS analysis	[67]
	200 μgL^−1^	500–25,000 μgL^−1^	drinking water	Bathocuproine	Smartphone-based digital image colorimetry	[60]
	4,000,000 µgL^−1^	4,000,000–20,000,000 µgL^−1^	Herbal supplements and cosmetics	5-Br-PAPS	Distance-based visual readout	[46]
	0.0051 µg	0.0051–0.2051 µg	Air	Dithiourea	Smartphone image analysis	[65]
	0.04584 µg	0.04584–0.4584 µg	Air	Selective colorimetric reagents	Smartphone-based grayscale analysis	[66]
Zn(II)	35.9 µgL^−1^	100–10,000 µgL^−1^	Urine	PAN	Digital image analysis	[38]
	2,000,000 µgL^−1^	2,000,000–10,000,000 µgL^−1^	Herbal supplements and cosmetics	5-Br-PAPS	Distance-based visual readout	[46]
	0.4 µg	-	Low explosive residue	Dithizone	Visual colorimetric screening	[40]
As(III)	10 µgL^−1^	1–50 µgL^−1^	Water	Au-TA-TG nanosensors	ImageJ-based color analysis	[47]
	5 μgL^−1^	5–10 μgL^−1^	Water	Functionalized AuNPs	Foldscope-assisted smartphone imaging	[62]
	0.5 µgL^−1^	0.5–1000 μgL^−1^	Urine	AgNPs	Visual or image analysis	[52]
Sb(III)	0.25 µg	-	Low explosive residue	Sodium sulfide	Visual colorimetric screening	[40]
Ba(II)	0.25 µg	-	Low explosive residue	Sodium rhodizonate	Visual colorimetric screening	[40]
Hg(II)	930 µgL^−1^	1000–30,000 µgL^−1^	Whitening cream	Dithizone	Distance-based readout	[42]
	1 µgL^−1^	1–1000 μgL^−1^	Water	CysA@AuNPs	UV-Vis analysis	[48]
	190 µgL^−1^	30–20,000 µgL^−1^	Water	Millethione	ImageJ-based RGB analysis	[57]
	200 µgL^−1^	200–12,040 µgL^−1^	Water	Dithizone	ImageJ-based image analysis	[58]
	1 μgL^−1^	50–7000 μgL^−1^	Water	AgNPs	RGB image analysis	[50]
	0.1 µgL^−1^	0.1–3 µgL^−1^	Food	AgNPs	RGB analysis	[51]
Pb(II)	0.0183 µM	0.1–10 µM	Water	CRISPR-Cas12a-based colorimetric probe	SmartIons app-based R/G analysis	[64]
	50,000 µgL^−1^	50,000–500,000 µgL^−1^, 600,000–1,000,000 µgL^−1^	Gunshot residue	Rhodizonate	Reaction band length detection	[43]
	756 μgL^−1^	3000–10,000 μgL^−1^	Wastewater	Sodium rhodizonate	Distance-based or RGB analysis	[44]
	0.05 µM	0.5–50 µM	Environmental	MTZ-AgNPs	Colorimetric readout	[49]
	0.1 µM	0.5–50 µM	Environmental	MTZ-AgNPs	distance-based detection	[49]
	152 µgL^−1^	100–700 µgL^−1^	Water	BSP-SP	Smartphone-based grayscale analysis	[39]
	0.25 µg	-	Low explosive residue	Sodium rhodizonate	Visual colorimetric screening	[40]

The table summarizes heavy metal ion detection methods, with metal ions listed according to the periodic table. -: No linear range was reported.

**Table 2:** Detection parameters of the indirect colorimetric method for heavy metal ion detection.

Heavy Metal Ion	Reported LOD	Linear Detection Range	Sample	Chromogenic Reagent	Readout	Reference
Al(III)	80 µgL^−1^	100–10,000 µgL^−1^	Environmental	Chrome azurol S	Smartphone-based image analysis	[68]
Cr(III)	3.0 µgL^−1^	7–200 µgL^−1^	Water	DPC	ImageJ-based green-channel analysis	[69]
Cr(VI)	100 µgL^−1^	200–17,000 µgL^−1^	Environmental	1,5-DPC	Smartphone-based image analysis	[68]
	2.0 µgL^−1^	10–200 µgL^−1^	Water	DPC	ImageJ-based green-channel analysis	[69]
Fe(III)	200 µgL^−1^	300–18,000 µgL^−1^	Environmental	1,10-Phenanthroline	Smartphone-based image analysis	[68]
Ni(II)	300 µgL^−1^	400–23,000 µgL^−1^	Environmental	DMG	Smartphone-based image analysis	[68]
Cu(II)	30 µgL^−1^	50–24,000 µgL^−1^	Environmental	Bathocuproine	Smartphone-based image analysis	[68]
Zn(II)	40 µgL^−1^	50–24,000 µgL^−1^	Environmental	Dithizone	Smartphone-based image analysis	[68]
Hg(II)	0.01 µM	0.025–0.5 µM	Water	TMB	Inverse color intensity	[71]
	20,000 μgL^−1^	50,000–350,000 μgL^−1^	Contaminated soil	Iodide-starch system	Image analysis	[72]
Pb(II)	0.0012 µM	0.01–1000 µM	Water	AuNPs	Image-based signal evaluation	[70]
	0.0007 µM	0.01–1000 µM	Water	AuNPs	Image-based signal evaluation	[70]

The table summarizes heavy metal ion detection methods, with metal ions listed according to the periodic table.

**Table 3:** Detection parameters of fluorescence methods for heavy metal ion detection.

Heavy Metal Ion	Reported LOD	Linear Detection Range	Sample	Method	Fluorescent Probe	Readout	Reference
Al(III)	2.5 µgL^−1^	2–54 µgL^−1^	Water	Fluorescence enhancement	Naphthalhydrazide derivative ligand	Distance-based fluorescence readout under UV light	[82]
	0.9 µgL^−1^	2–24 µgL^−1^	Water	Fluorescence enhancement	Naphthalhydrazide derivative ligand	Distance-based fluorescence readout under UV light	[82]
Fe(III)	0.019 µM	0.2–160 µM	Water	Fluorescence ratiometric	FMOF-5@TC nanocomposite	Smartphone-based ratiometric fluorescence imaging	[87]
Cu(II)	5 µM	0.1–500 µM	Water	Fluorescence quenching	Au-BSA NCs	Distance-based visual readout under UV light	[73]
	0.035 µgL^−1^	0.11–58.0 µgL^−1^	Water	Fluorescence quenching	Cu(II)-IIP-modified CdTe QDs	Fluorescence intensity readout under UV light	[78]
	100 µM	-	Water	Fluorescence quenching	NCDs	Fluorescence intensity readout	[76]
	0.25 µgL^−1^	-	Water	Fluorescence quenching	CdTe QDs	Fluorescence readout under LED excitation	[81]
	0.027 µM	0.1–80 µM	Water	Fluorescence ratiometric	FMOF-5@TC nanocomposite	Smartphone-based ratiometric fluorescence imaging	[87]
As(III)	0.00096 µM	0.001–1 µM	Water	Fluorescence enhancement	Q-Apt-34/C-FAM-11 duplex	Smartphone-based fluorescence imaging	[85]
	0.00165 µM	0.02–0.5 µM	Food	Fluorescence quenching	As(III)-specific aptamer	Smartphone-based fluorescence imaging	[84]
Ag(I)	0.047 µM	0–1.75 µM	Food	Fluorescence quenching	Cy5-labeled ssDNA/GO	Fluorescence intensity readout	[75]
Cd(II)	0.245 μgL^−1^	1–70 μgL^−1^	Water	Fluorescence quenching	Cd(II)-IIP-modified ZnSe QDs	Fluorescence intensity readout under UV light	[80]
	0.17 µgL^−1^	-	Water	Fluorescence quenching	CdTe QDs	Fluorescence readout under LED excitation	[81]
	0.00204 µM	0.02–0.2 µM	Food	Fluorescence enhancement	Cd(II)-specific aptamer	Smartphone-based fluorescence imaging	[84]
	0.00856 µM	0.01–0.2 µM	Vegetables	Fluorescence enhancement	FAM-labeled Cd(II) aptamer–BHQ duplex	Smartphone imaging with deep learning analysis	[86]
Hg(II)	0.121 µM	0–3 µM	Food	Fluorescence quenching	Cy5-labeled ssDNA/GO	Fluorescence intensity readout	[75]
	2.83 μgL^−1^	0 µgL^−1^–100 μgL^−1^	Water	Fluorescence quenching	CdTe QDs-SiO_2_	Smartphone-based fluorescence imaging under UV light	[74]
	0.056 µgL^−1^	0.26–34.0 µgL^−1^	Water	Fluorescence quenching	Hg(II)-IIP-modified CdTe QDs	Fluorescence intensity readout under UV light	[78]
	0.18 μgL^−1^	0.5–20 μgL^−1^	Water	Fluorescence quenching	Hg(II)-IIP-modified CdTe QDs on cloth	Fluorescence intensity readout under UV light	[79]
	100 µM	-	Water	Fluorescence quenching	NCDs	Fluorescence intensity readout	[76]
	0.36 µgL^−1^	-	Water	Fluorescence quenching	CdTe QDs	Fluorescence readout under LED excitation	[81]
	1.54 µgL^−1^	1–100 µgL^−1^	Water	Fluorescence quenching	Blue fluorescent CNPs	Smartphone-based fluorescence imaging under UV light	[77]
	0.0017 µM	0.01–1 µM	Food	Fluorescence quenching	T-rich aptamer-based system	Smartphone-based fluorescence imaging	[84]
Pb(II)	0.07 μgL^−1^	0.5–20 μgL^−1^	Water	Fluorescence quenching	Pb(II)-IIP-modified CdTe QDs on cloth	Fluorescence intensity readout under UV light	[79]
	0.335 μgL^−1^	1–60 μgL^−1^	Water	Fluorescence quenching	Pb(II)-IIP-modified ZnSe QDs	Fluorescence intensity readout under UV light	[80]
	0.23 µgL^−1^	-	Water	Fluorescence quenching	CdTe QDs	Fluorescence readout under LED excitation	[81]
	0.001 µM	0.01–0.1µM	Water	Fluorescence quenching	G-quadruplex–iridium(III) complex system	Smartphone-based fluorescence imaging	[83]
	0.0042 µM	0.02–0.5 µM	Food	Fluorescence quenching	GR-5 DNAzyme-based system	Smartphone-based fluorescence imaging	[84]

The table summarizes heavy metal ion detection methods, with metal ions listed according to the periodic table. -: No linear range was reported.

**Table 4:** Detection parameters of electrochemical methods for heavy metal ion detection.

Heavy Metal Ion	Reported LOD	Linear Detection Range	Sample	Method	Electrode or Sensing Element	Readout	Reference
Fe(II)	0.5 μgL^−1^	0.5–400.0 μgL^−1^	Aerosol	Voltammetry	Bi- and Nafion-modified WE with 1,10-phenanthroline	Stripping peak current readout	[102]
Ni(II)	1 μgL^−1^	0.5–200.0 μgL^−1^	Aerosol	Voltammetry	Bi- and Nafion-modified WE with DMG	Stripping peak current readout	[102]
Cu(II)	1 μgL^−1^	1–400.0 μgL^−1^	Aerosol	Voltammetry	Bi- and Nafion-modified WE	Stripping peak current readout	[102]
Zn(II)	1.1 μgL^−1^	5–40 μgL^−1^	Environmental	Voltammetry	Sn thin-film WE	Stripping peak current readout	[95]
As(III)	20 μgL^−1^	100–1500 μgL^−1^	Rice	Voltammetry	AuNP-modified BDD electrode	Stripping peak current readout	[97]
Cd(II)	100 µmolL^−1^	100–10^4.6^ µmolL^−1^	Environmental	Potentiometric	Cd(II)-ISE	Potential readout	[90]
	10 μmolL^−1^	10–10^4.7^ μmolL^−1^	Water	Potentiometric	Cd(II)-ISE	Potential readout	[91]
	0.05 μgL^−1^	1–100 μgL^−1^	Water	Voltammetry	IIPs@paper and rGO-modified pSPCE	Stripping peak current readout	[101]
	25 μgL^−1^	25–200 μgL^−1^	Water	Voltammetry	Bismuth-film-modified BDDPE	Stripping peak current readout	[98]
	1.2 μgL^−1^	5–500 μgL^−1^	Water	Voltammetry	Graphite foil electrode	Anodic peak current readout	[94]
	0.5698 µg L^−1^	5–100 µgL^−1^	Water	Voltammetry	NG-modified pSPCE	Stripping peak current readout	[99]
	0.9 μgL^−1^	5–40 μgL^−1^	Environmental	Voltammetry	Sn thin-film WE	Stripping peak current readout	[95]
	52.8 μgL^−1^	10–110 μgL^−1^	Water	Voltammetry	Screen-printed carbon WE	Stripping peak current readout	[96]
	0.00002331 µmolL^−1^	0.0001–1 µmolL^−1^	Food	Voltammetry	AuNP-modified WE with Cd(II) aptamer	Signal-off current readout	[103]
	0.00000084 µmolL^−1^	0.00001–1 µmolL^−1^	Crab meat	voltammetry	MB-labeled Cd(II) aptamer–CP duplex	Signal-off current readout	[104]
	0.5 μgL^−1^	0.5–400.0 μgL^−1^	Aerosol	Voltammetry	Bi- and Nafion-modified WE	Stripping peak current readout	[102]
Hg(II)	0.00000163 µmolL^−1^	0.00001–1 µmolL^−1^	Crab meat	voltammetry	MB-labeled Hg(II) aptamer–CP duplex	Signal-off current readout	[104]
	0.2565 µg L^−1^	5–100 µgL^−1^	Water	Voltammetry	NG-modified pSPCE	Stripping peak current readout	[99]
Pb(II)	10 µmolL^−1^	10–10^3.8^ µmolL^−1^	Water	Potentiometric	Pb(II)-ISE	Potential readout	[88]
	10^0.6^ µmolL^−1^	10–10^3.83^ µmolL^−1^	Water	Potentiometric	Pb(II)-ISM	Potential readout	[89]
	10 µmolL^−1^	10–10^4^ µmolL^−1^	Environmental	Potentiometric	Metal-modified Pb(II)-ISE	Potential readout	[92]
	10 µmolL^−1^	10^4.65^–10 µmolL^−1^	Environmental	Potentiometric	Pb(II)-ISE	Potential readout	[90]
	10 μmolL^−1^	10–10^3.8^ μmolL^−1^	Water	Potentiometric	Pb(II)-ISE	Potential readout	[91]
	0.016 μmolL^−1^	0.050–0.5 μmolL^−1^	Water	Voltammetry	LPSE	Stripping peak current readout	[93]
	9 μgL^−1^	10–500 μgL^−1^	Urine	Voltammetry	Gold-plated plastic WE with (NH_4_)_2_SO_4_-modified paper	Stripping peak current readout	[100]
	1 μgL^−1^	1–200 μgL^−1^	Water	Voltammetry	Bismuth-film-modified BDDPE	Stripping peak current readout	[98]
	1.8 μgL^−1^	5–100 μgL^−1^	Water	Voltammetry	Graphite foil electrode	Anodic peak current readout	[94]
	0.4024 µgL^−1^	5–100 µg L^−1^	Water	Voltammetry	NG-modified pSPCE	Stripping peak current readout	[99]
	20.4 μgL^−1^	10–110 μgL^−1^	Water	Voltammetry	Screen-printed carbon WE	Stripping peak current readout	[96]
	0.00004623 µmolL^−1^	0.0001–1 µmolL^−1^	Food	voltammetry	AuNP-modified WE with Pb(II) aptamer	Signal-off current readout	[103]
	0.00000155 µmolL^−1^	0.00001–1 µmolL^−1^	Crab meat	voltammetry	MB-labeled Pb(II) aptamer–CP duplex	Signal-off current readout	[104]
	0.5 μgL^−1^	0.5–400.0 μgL^−1^	Aerosol	Voltammetry	Bi- and Nafion-modified WE	Stripping peak current readout	[102]

The table summarizes heavy metal ion detection methods, with metal ions listed according to the periodic table.

**Table 5:** Detection parameters of dual-detection systems and other µPAD-based methods for heavy metal ion detection.

Heavy Metal Ion	Reported LOD	Linear Detection Range	Sample	Method	Detection Reagent, Material, or Sensing Element	Readout	Reference
Cr(III)	20 µgL^−1^	50–1000 µgL^−1^	Water	Chemiluminescence method	Luminol–hydrogen peroxide system	PMT-based chemiluminescence intensity readout	[109]
	24.5 μgL^−1^	25–35,000 μgL^−1^, 50,000–500,000 μgL^−1^	Water	Chemiluminescence method	Luminol–H_2_O_2_ system	CCD-based chemiluminescence intensity readout	[110]
Cr(VI)	10 µgL^−1^	50–3000 µgL^−1^	Water	Colorimetric and electrochemical methods	1,5-DPC and three-electrode system	Smartphone colorimetric readout and LSV current readout	[105]
Total Cr	60 μgL^−1^	200–3000 μgL^−1^	Water	Colorimetric and electrochemical methods	Ce(IV), 1,5-DPC, and three-electrode system	Smartphone colorimetric readout and LSV current readout	[105]
Fe(II)	100 µgL^−1^	1000–20,000 µgL^−1^	Water	Colorimetric and electrochemical methods	1,10-Phenanthroline	Colorimetric image readout	[108]
Ni(II)	300 µgL^−1^	1000–50,000 µgL^−1^	Water	Colorimetric and electrochemical methods	Dimethylethylenediamine	Colorimetric image readout	[108]
Cu(II)	4.12 μgL^−1^	10–350 μgL^−1^	Environmental and food	Colorimetric and electrochemical methods	Thiosulfate and silver nanoplatelets	Visual and digital image colorimetric readout	[106]
	8.51 μgL^−1^	50–100 μgL^−1^	Mineral Water	Colorimetric and electrochemical methods	TA- and DNS-functionalized AuNPs	RGB sensor-based colorimetric readout	[107]
	200 µgL^−1^	1000–25,000 µgL^−1^	Water	Colorimetric and electrochemical methods	Pyridinoxazole	Colorimetric image readout	[108]
Zn(II)	10.5 μgL^−1^	100–1400 μgL^−1^	Water	Colorimetric and electrochemical methods	Carbon nanotube-modified graphite WE	SWASV stripping peak current readout	[108]
Cd(II)	0.1 μgL^−1^	0.5–70 μgL^−1^	Environmental and food	Colorimetric and electrochemical methods	Bismuth-modified BDD electrode	ASV stripping peak current readout	[106]
	1.588 μgL^−1^	0–100 μgL^−1^	Mineral Water	Colorimetric and electrochemical methods	Bismuth-film-modified WE	SWASV stripping peak current readout	[107]
	1.3 μgL^−1^	10–1400 μgL^−1^	Water	Colorimetric and electrochemical methods	Carbon nanotube-modified graphite WE	SWASV stripping peak current readout	[108]
	3 µgL^−1^	0–150 µgL^−1^	Cucumber plants	LIBS	PMSE-LIBS	LIBS emission intensity readout	[111]
Pb(II)	0.1 µgL^−1^	0.5–70 µgL^−1^	Environmental and food	Colorimetric and electrochemical methods	Bismuth-modified BDD electrode	ASV stripping peak current readout	[106]
	1.42 μgL^−1^	0–100 μgL^−1^	Mineral Water	Colorimetric and electrochemical methods	Bismuth-film-modified WE	SWASV stripping peak current readout	[107]
	0.9 μgL^−1^	10–1400 μgL^−1^	Water	Colorimetric and electrochemical methods	Carbon nanotube-modified graphite WE with bismuth film	SWASV stripping peak current readout	[108]
	5 µgL^−1^	0–150 µgL^−1^	cucumber plants	LIBS	CuNP-modified enrichment filter paper	LIBS emission intensity readout	[111]

The table summarizes heavy metal ion detection methods, with metal ions listed according to the periodic table. LIBS: Laser-Induced Breakdown spectroscopy.

## Data Availability

No new data were created or analyzed in this study. Data sharing is not applicable to this article.

## Abbreviations

The following abbreviations are used in this manuscript:

ICP-MS	Inductively coupled plasma mass spectrometry
ICP-AES	Inductively coupled plasma atomic emission spectroscopy
AAS	Atomic absorption spectroscopy
UV-Vis	Ultraviolet-visible spectroscopy
µPADs	Paper-based microfluidic analytical devices
POCT	Point-of-care testing
2D	Two-dimensional
3D	Three-dimensional
ePADs	Electrochemical paper-based analytical devices
1,5-DPC	1,5-Diphenylcarbazide
PAN	1-(2-Pyridylazo)-2-naphthol
RGB	Red, green, and blue
SPE	Solid-phase extraction
EDM	Euclidean distance method
BSP-SP	1-Benzyl-3,3-dimethylspiro[indoline-2,3′-naphtho [2,1-b][1,4]oxazine]
UV	Ultraviolet
DPCO	1,5-Diphenylcarbazone
PDMS	Polydimethylsiloxane
DDTC	Sodium diethyldithiocarbamate
5-Br-PAPS	2-(5-Bromo-2-pyridylazo)-5-[N-propyl-N-(3-sulfopropyl)amino]phenol
AuNPs	Gold nanoparticles
AgNPs	Silver nanoparticles
LSPR	Localized surface plasmon resonance
CysA@AuNPs	Cysteine-modified gold nanoparticles
MTZ-AgNPs	Metronidazole-functionalized silver nanoparticles
PVC	Polyvinyl chloride
DMG	Dimethylglyoxime
PAR	4-(2-Pyridylazo)resorcinol
LED	Light-emitting diode
CRISPR-Cas12a	Clustered regularly interspaced short palindromic repeats-associated protein 12a
ssDNA	Single-stranded DNA
R/G	Red/green ratio
UAV	Unmanned aerial vehicle
LIBS	Laser-induced breakdown spectroscopy
LaPAD	LIBS-assisted paper-based microfluidic analytical device
TMB	3,3′,5,5′-Tetramethylbenzidine
SD-µPAD	Suspending-droplet mode paper-based microfluidic analytical device
FAM	Fluorescein amidite
BHQ	Black hole quencher
FMOF	Fluorescent metal–organic framework
TC	Tetracycline
NaF	Sodium fluoride
WE	Working electrode
RE	Reference electrode
CE	Counter electrode
ISM	Ion-selective membrane
ISEs	Ion-selective electrodes
PEDOT:PSS	Poly(3,4-ethylenedioxythiophene)(styrenesulfonate)
LPSE	Laminated polycaprolactone/paper/silver electrode
PCL	Polycaprolactone
SWASV	Square-wave anodic stripping voltammetry
BDD	Boron-doped diamond
BDDPE	Boron-doped diamond paste electrode
SWV	Square-wave voltammetry
NG	Nitrogen-doped graphene
DPV	Differential pulse voltammetry
ASV	Anodic stripping voltammetry
IIPs@paper	Ion-imprinted polymer-modified paper
rGO	Reduced graphene oxide
SWCSV	Square-wave cathodic stripping voltammetry
MB	Methylene blue
Fc	Ferrocene
LSV	Linear sweep voltammetry
PMT	Photomultiplier tube
CCD	Charge-coupled device
